# An Interpretable SERS–AI Platform for Rapid and Quantitative Diagnosis of Polymicrobial UTIs: Powered by Positively Charged Plasmonic Nanoparticles and Attention‐Based Deep Learning

**DOI:** 10.1002/advs.202513502

**Published:** 2025-09-24

**Authors:** Zhonghua Shen, Linguo Xie, Yuwei Hou, Junjie Liang, Yuchi Jia, Haipeng Zhang, Zhenli Sun, Jingjing Du, Zeying He, Chunyu Liu, Wenjing Liu

**Affiliations:** ^1^ Key Laboratory for Environmental Factors Control of Agro‐product Quality Safety Agro‐Environmental Protection Institute Ministry of Agriculture and Rural Affairs Tianjin 300191 China; ^2^ Department of Urology Tianjin Institute of Urology The Second Hospital of Tianjin Medical University Tianjin 300211 China; ^3^ Department of Radiology Tianjin Beichen Hospital Tianjin 300400 China; ^4^ MOE Key Laboratory of Resources and Environmental System Optimization College of Environmental Science and Engineering North China Electric Power University Beijing 102206 China; ^5^ State Key Laboratory of Environmental Chemistry and Ecotoxicology Research Center for Eco‐Environmental Sciences Chinese Academy of Sciences Beijing 100085 China

**Keywords:** convolutional neural network (CNN), convolutional block attention module (CBAM), mixed bacteria, proportion, surface‐enhanced Raman spectroscopy (SERS)

## Abstract

Polymicrobial urinary tract infections (UTIs) present diagnostic challenges due to overlapping symptoms and limitations of conventional methods. Although SERS and AI have shown potential for microbial diagnostics, existing approaches lack reproducibility, quantification capability, and interpretability—especially in complex clinical samples. Here, a label‐free, interpretable SERS‐AI platform for rapid identification and quantification of mixed urinary tract pathogens is proposed. A plasmonic substrate is engineered by combining Au@Ag core–shell nanoparticles with a positively charged bPEI surface, enabling electrostatic bacterial capture and stable SERS signal generation across diverse microbial mixtures. A convolutional neural network (CNN) enhanced with a convolutional block attention module (CBAM) to enable both accurate classification (95.8%, AUC  =  0.9774) and reliable bacterial proportion prediction (R^2^  =  0.9112), surpassing traditional models, is developed. Importantly, the attention mechanism offers mechanistic interpretability, highlighting biologically relevant spectral features related to nucleic acids, proteins, and virulence factors. Validation with clinical urine samples demonstrates strong predictive performance (accuracy = 86.9%, R^2^ = 0.8626), supporting real‐world applicability. Overall, this work not only delivers a high‐throughput and explainable framework for polymicrobial diagnostics, but also contributes to the mechanistic understanding of Raman‐based microbial phenotyping, paving the way for clinical deployment and microbiome‐informed interventions.

## Graphical abstract

This work introduces a label‐free SERS‐AI platform for rapid and interpretable diagnosis of polymicrobial urinary tract infections. By integrating Au@Ag@bPEI substrates with a CNN‐CBAM model, the system enables accurate bacterial identification and proportion prediction, while highlighting biologically relevant Raman features. Clinical validation confirms strong performance, supporting translational potential for real‐world diagnostics.

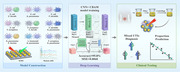

## 1 Introduction

Urinary tract infections (UTIs) are among the most common infectious pathogens encountered in clinical practice,^[^ ^1^, ^2^ ^]^ typically caused by bacteria. Mixed infections represent an important and clinically significant subset. Notably, the incidence of mixed infections is considerably higher in complicated UTIs and catheter‐associated UTIs, reaching rates as high as 30–86% in some reports.^[^ ^3^, ^4^ ^]^ These infections frequently involve two distinct pathogens and are often associated with complex pathophysiological interactions, such as synergistic interactions between co‐infecting organisms.^[^ ^5^ ^]^ Such interactions can result in enhanced virulence, increased biofilm formation, and elevated resistance to antimicrobial therapies. The coexistence of multiple pathogens complicates diagnosis and treatment selection; if not all organisms are effectively targeted, residual bacteria may persist, potentially leading to treatment failure, infection recurrence, or chronic disease. These risks are particularly pronounced in immunocompromised patients.^[^ ^6^ ^]^ Inadequate treatment of mixed UTIs may promote the emergence of antibiotic resistance, further complicating clinical management.^[^ ^7^ ^]^ Therefore, rapid and accurate identification of polymicrobial UTIs is essential for guiding effective antibiotic therapy and avoiding delayed or inadequate treatment.

Conventional diagnostic approaches for UTIs—including urine culture,^[^ ^8^ ^]^ test strips,^[^ ^9^, ^10^ ^]^ microscopy,^[^ ^11^, ^12^ ^]^ and polymerase chain reaction (PCR)^[^ ^13^, ^14^ ^]^—are constrained by long turnaround times, low sensitivity, and insufficient ability to detect and differentiate multiple pathogens simultaneously. These limitations highlight the urgent need for faster and more precise diagnostic strategies tailored to the complexity of clinical scenarios. Surface‐enhanced Raman spectroscopy (SERS) has emerged as a powerful analytical technique due to its high sensitivity and ability to capture molecular‐level information, making it particularly suitable for bacterial detection.^[^ ^15^, ^16^, ^17^ ^]^ By amplifying Raman signals, SERS can capture distinctive spectral signatures of bacterial components, including proteins, nucleic acids, and lipids, enabling bacterial species‐level identification.^[^ ^18^, ^19^, ^20^ ^]^ Compared to traditional methods, SERS offers clear advantages for analyzing complex biological samples.^[^ ^21^, ^22^ ^]^

Previous studies have demonstrated the potential of Raman spectroscopy for identifying UTI pathogens, including in polymicrobial samples, as a rapid alternative to conventional microbiological techniques. For instance, Aubrechtová et al.^[^ ^23^ ^]^ developed a PCA‐LDA model to classify bacterial Gram types and families, achieving 75% accuracy in detecting mixed infections in artificial mixtures. Similarly, Yogesha et al.^[^ ^24^ ^]^ applied micro‐Raman spectroscopy combined with multivariate methods such as PLS‐DA and SVM, reaching up to 89% accuracy in mixed cultures. While these studies represent important advances, several limitations hinder their clinical applicability: Limited by low bacterial diversity, reliance on synthetic mixtures, lack of proportion prediction, the bacterial proportion can significantly influence infection severity, pathogenic dominance, and antibiotic response. Finally, the outdated machine learning methods hindering clinical applicability and accurate spectral analysis.

Convolutional neural network (CNN) integrated with convolutional block attention module (CBAM) has shown strong capabilities in medical image analysis^[^ ^25^ ^]^ and tumor classification^[^ ^26^ ^]^ due to their enhanced feature extraction and interpretability via attention mechanisms. CBAM adaptively emphasizes key spectral features while suppressing noise, making it well suited for Raman data with complex, overlapping signals. This is especially beneficial in mixed UTI detection, where coexisting pathogens produce convoluted spectra. However, the use of CNN+CBAM in Raman‐based bacterial identification—particularly in polymicrobial clinical samples—has not yet been reported, highlighting a key innovation of this study.

To address these challenges, we present a novel SERS‐based diagnostic framework that integrates a positively charged Au@Ag@bPEI substrate with a deep learning architecture combining CNN and CBAM. This dual‐task model enables simultaneous classification of bacterial species and quantification of their relative abundances, offering comprehensive information essential for guiding personalized antimicrobial treatment. The framework achieved high diagnostic performance, with 95.8% classification accuracy and an R^2^ of 0.9112 for bacterial proportion prediction, and demonstrated reliable generalizability in clinical urine samples. To the best of our knowledge, this is the first application of the CNN+CBAM architecture for interpreting SERS spectra in the context of polymicrobial UTIs. In addition, Attention‐based visualizations revealed consistent focus on biologically meaningful spectral features, supporting interpretable and mechanism‐driven identification of mixed pathogens. Beyond UTIs, this interpretable and scalable approach establishes a mechanistic and computational foundation for Raman‐based polymicrobial detection, with broad potential applications in clinical diagnostics, infection surveillance, and microbiome research.

## 2 Results and Discussion

### 2.1 Mixed Bacteria in UTIs

A retrospective analysis was conducted on the distribution of mixed UTIs at the Second Hospital of Tianjin Medical University between January 2021 and January 2023. A total of 264 patients with culture‐confirmed mixed infections were identified (Figure S1, Supporting Information). Among these, nine bacterial combinations with at least 10 documented cases were selected for further analysis.

The most prevalent pairing was *Escherichia coli* (*E. coli*) and *Enterococcus faecalis* (*E. faecalis*) (35 cases), followed by *E. coli* and *Klebsiella pneumoniae* (*K. pneumoniae*) (30 cases). Other notable combinations included *Acinetobacter baumannii* (*A. baumannii*) with *K. pneumoniae*, *E. coli* with *Pseudomonas aeruginosa* (*P. aeruginosa*), *E. coli* with *Proteus mirabilis* (*P. mirabilis*), *Enterococcus faecium* (*E. faecium*) with *K. pneumoniae*, *E. faecalis* with *P. aeruginosa*, *P. mirabilis* with *K. pneumoniae*, and *P. aeruginosa* with *K. pneumoniae*.

Overall, *E. coli* was the most frequently detected pathogen in mixed infections, commonly appearing alongside other species. *K. pneumoniae* and *E. faecalis* also appeared frequently, underscoring their significant roles in the polymicrobial UTI cases.

### 2.2 Characterization and Performance of SERS Substrate

**Figure 1:**
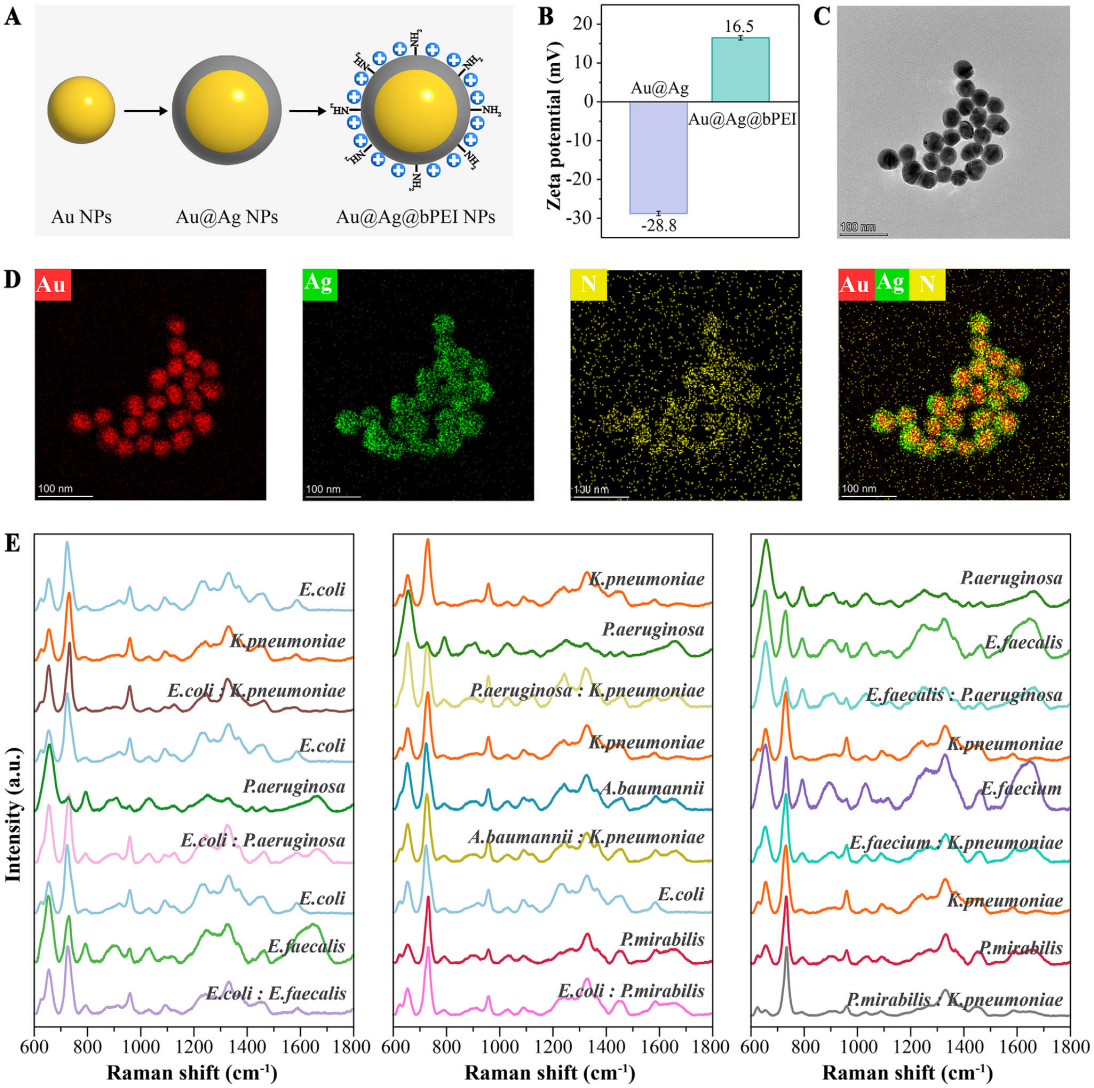
Characterization of Au@Ag@bPEI nanoparticles and Raman spectral fingerprints. A) Schematic illustration of the stepwise synthesis of Au@Ag@bPEI nanoparticles (NPs), including the formation of Au NPs, Au@Ag core–shell NPs, and subsequent surface modification with bPEI. B) Zeta potential analysis showing the surface charge transition from −28.8 mV (Au@Ag NPs) to +16.5 mV after bPEI modification. C) HR‐TEM image confirming the structure of Au@Ag@bPEI NPs. D) Elemental mapping images obtained via energy‐dispersive X‐ray spectroscopy (EDS) show the spatial distribution of Au (red), Ag (green), and N (yellow), confirming the core–shell structure and successful bPEI modification. E) Raman spectral fingerprints of single and mixed pathogenic bacterial species across the 600–1800 cm^−1^ range.

To achieve stable and highly sensitive SERS substrates for detecting bacteria in complex biological settings, core–shell Au@Ag nanoparticles were chosen. This choice was based on the need for both sensitivity and stability when identifying bacteria from complex urine samples. Silver provides excellent plasmonic enhancement essential for identifying low‐abundance bacterial signatures. However, bare Ag nanoparticles are prone to oxidation and instability, which compromise reproducibility in clinical use. By constructing a silver shell over a gold core, the Au@Ag structure exposes the SERS‐active silver surface directly to the analytes while the gold core serves as a stable scaffold that enhances monodispersity and mitigates silver degradation.^[^ ^27^, ^28^, ^29^ ^]^ In contrast, an Ag@Au configuration would shield silver's hotspots beneath a gold layer, diminishing sensitivity—an unacceptable trade‐off when analyzing trace bacterial signals in urine. Thus, Au@Ag offers the optimal balance, ensuring strong enhancement, reproducibility, and robustness needed for reliable SERS‐based bacterial detection in real clinical samples. Given the negatively charged nature of bacterial surfaces,^[^ ^30^ ^]^ we synthesized branched polyethyleneimine (bPEI)‐functionalized Au@Ag NPs to impart a positive surface charge, thereby promoting electrostatic interactions and improving the binding affinity between the nanoparticles and bacterial cells. The synthesis process of the Au@Ag@bPEI nanoparticles (NPs) was shown in **Figure** **1**A, briefly, Au@Ag@bPEI NPs were synthesized by first preparing Au NPs, then coating them with a silver shell to form Au@Ag cores, followed by surface modification with bPEI to enhance stability and bacterial binding through positive charge. To demonstrate the impact of bPEI modification on the surface charge of the nanomaterials, Zeta potential measurements confirmed successful modification of surface charge: the Zeta potential of Au@Ag NPs showed −28.8 mV, while that of Au@Ag@bPEI NPs exhibited 16.5 mV (Figure 1B).

HR‐TEM images revealed the uniform spherical core–shell structure for Au@Ag@bPEI NPs (Figure 1C,D). Elemental mapping displayed that bPEI was evenly distributed on the surface of Au@Ag NPs (Figure 1D). For Au@Ag@bPEI NPs, the peaks at 83.3 and 87.0 eV were ascribed to the 4f_7/2_ and 4f_5/2_ of Au(0),^[^ ^31^ ^]^ and the peaks at 367.2 and 373.0 eV, corresponding to the 3d_5/2_ and 3d_3/2_ levels of Ag(0),^[^ ^32^ ^]^ In addition, the high‐resolution N 1s XPS spectrum displayed two peaks at 398.5 and 400.0 eV, corresponding to neutral amine groups (‐NH_2_) in bPEI and nitrogen coordinated with silver (N‐Au/Ag), respectively, indicating the formation of N‐Au/Ag coordination bonds^[^ ^33^ ^]^ (Figure S2, Supporting Information).

The detection limit for R6G using Au@Ag@bPEI NPs was 10^−9^ mmol L^−1^ (Figure S3, Supporting Information). The positively charged Au@Ag@bPEI NPs, as an SERS substrate, detected *E. coli* as low as 5×10^2^ CFU mL^−1^. A clear linear relationship between Raman intensity and *E. coli* concentration demonstrated that the substrate is suitable for reliable quantitative detection (Figure S6, Supporting Information).

### 2.3 SERS Spectral Measurement of Mixed Bacteria

The Au@Ag@bPEI NPs substrate was applied for the detection of mixed bacterial samples (Figure 1E), and peak assignments are listed in Table S1 (Supporting Information). Common Raman peaks among single bacterial species were mainly distributed in regions related to proteins (644–657,^[^ ^34^ ^]^ 1030–1032,^[^ ^35^, ^36^, ^37^ ^]^ 1325–1336 cm^−1^^[^ ^38^ ^]^), amino acids (959 cm^−1^),^[^ ^39^ ^]^ carbohydrates (885, 898 cm^−1^),^[^ ^40^ ^]^ and nucleic acids (794 cm^−1^, guanine).^[^ ^41^ ^]^ Species‐specific peaks were also observed: *E. faecalis* (904 cm^−1^), *P. aeruginosa* (655 cm^−1^), *P. mirabilis* (732 and 1331 cm^−1^). *A. baumannii, K. pneumoniae, and E. coli* shared peaks ≈644–657 cm^−1^, 724–738 cm^−1^, and 1242–1331 cm^−1^.

Mixed bacterial spectra exhibited both shared and distinctive features compared to their single counterparts. In *E. coli* + *P. aeruginosa* mixtures, peaks at 655 cm^−1^ and 1242–1245 cm^−1^ were enhanced. Similar spectral interactions were observed in mixtures involving *E. faecalis*, *P. mirabilis*, and *A. baumannii*. However, combinations such as *K. pneumoniae* + *E. coli* produced spectra nearly identical to the individual bacteria. These spectral phenomena highlighted the challenge of identifying mixed infections based solely on Raman peak positions.

### 2.4 CNN+CBAM‐Based Classification of Mixed Bacteria

**Figure 2:**
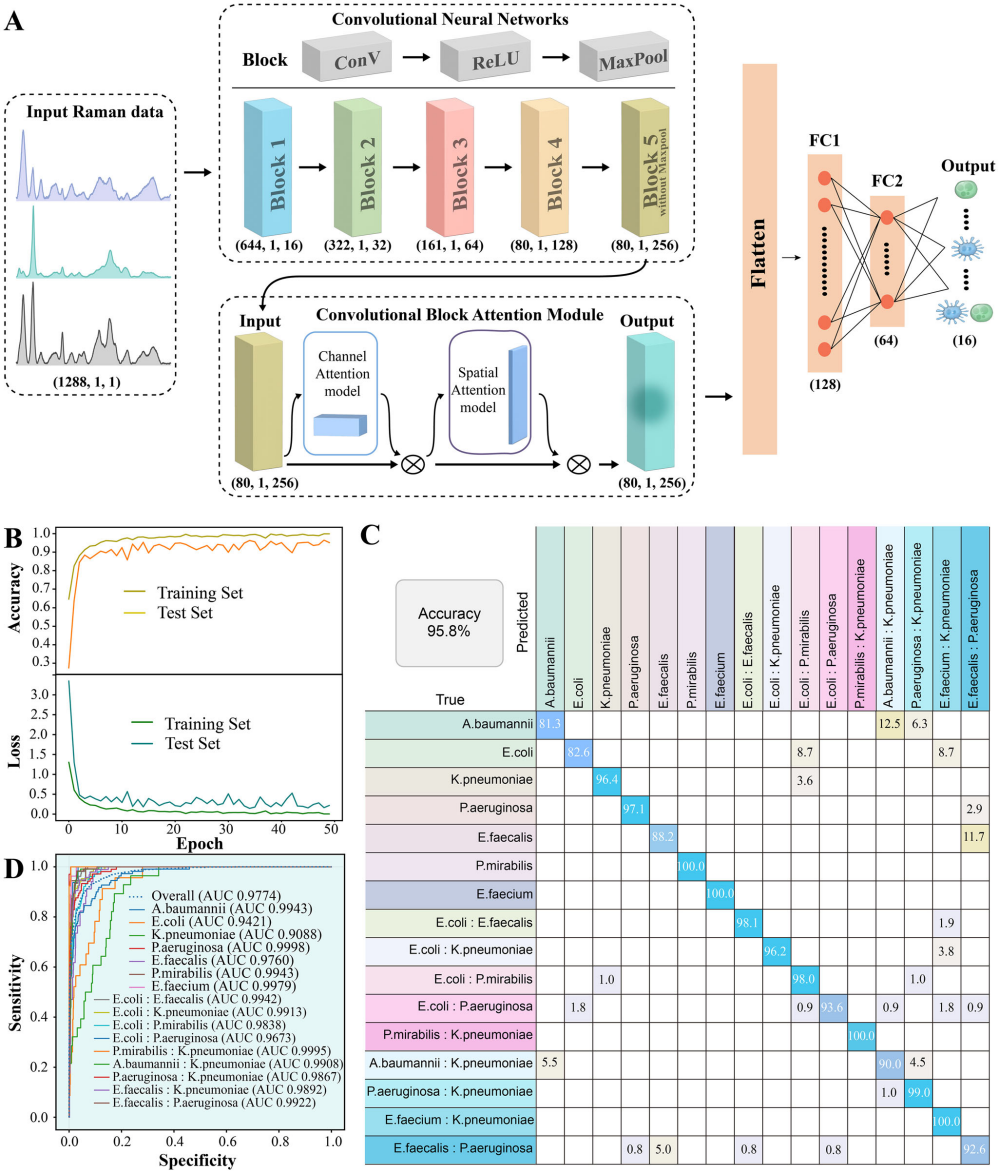
CNN+CBAM‐based classification of mixed pathogenic bacteria using Raman spectra. A) Schematic of the convolutional neural network (CNN) with a Convolutional Block Attention Module (CBAM) designed to classify Raman spectra. B) Model training and test performance curves: accuracy (top) and loss (bottom) across 50 epochs. C) Confusion matrix for the test set predictions with an overall classification accuracy of 95.8%. D) Receiver operating characteristic (ROC) curves and area under the curve (AUC) for all classes, including the macro average and each individual category.

A CNN+CBAM deep learning model was employed for accurate classification of mixed bacterial species from SERS data (**Figure** **2**A). Raman spectral data were first processed through convolutional layers for hierarchical feature extraction, followed by CBAM attention modules that enhanced the representation of critical spectral features. These refined features were then passed through fully connected layers to generate classification outputs for 16 bacterial categories.

As illustrated in Figure 2B, both training and test accuracies steadily increased and stabilized over the course of training, indicating effective learning and good generalization. The loss function rapidly declined during the initial epochs and subsequently plateaued, suggesting convergence of the model. Similar trends were observed in the remaining four cross‐validation folds (Figure S7, Supporting Information), demonstrating the model's robustness and consistency across different data splits. These results confirm that the CNN+CBAM model effectively extracts diagnostic features from SERS spectra and accurately classifies bacterial species. The model achieved an overall accuracy of 95.8% (Figure 2C), with near‐perfect classification for most strains, including 100% accuracy for *P. mirabilis*, *E. faecium*, and their mixed infections with *K. pneumoniae*. These results demonstrate the model's strong discriminative ability, especially for certain individual and mixed bacterial types. To evaluate overall classification performance, receiver operating characteristic (ROC) curves and area under the curve (AUC) values were calculated (Figure 2D). The model achieved an average AUC of 0.9774, reflecting excellent performance in distinguishing between individual and mixed bacterial categories. Notably, all bacterial groups exhibited AUC values greater than 0.9000, further supporting the robustness of the model.

A performance comparison between CNN+CBAM and three alternative models—including standalone CNN, Extreme Gradient Boosting (XGBoost), and Random Forest (RF)—is presented in **Table** **1**. The CNN+CBAM model achieved the highest performance, with 95.8% accuracy, 94.1% precision, 94.6% recall, and an F1‐score of 94.2%. The CNN model without CBAM showed slightly lower performance, particularly in recall (85.8%) and F1‐score (87.8%), despite maintaining high precision. In contrast, traditional machine learning models (XGBoost and RF) exhibited inferior performance across all metrics. Detailed CNN+CBAM results were provided in Figure S8 (Supporting Information).

**Table 1:** Comparison of CNN + CBAM performance with three machine learning algorithms for predicting individual and mixed bacterial.

Algorithm	Accuracy	Precision	Recall	F1‐score
CNN+CBAM	0.958	0.941	0.946	0.942
CNN	0.926	0.941	0.858	0.878
XGBoost	0.918	0.890	0.872	0.879
RF	0.833	0.865	0.744	0.776

CNN: Convolutional Neural Networks; BAM: Convolutional Block Attention Module; XGBoost: eXtreme Gradient Boosting; RF: Random Forest.

### 2.5 Attention‐Based Interpretability

**Figure 3:**
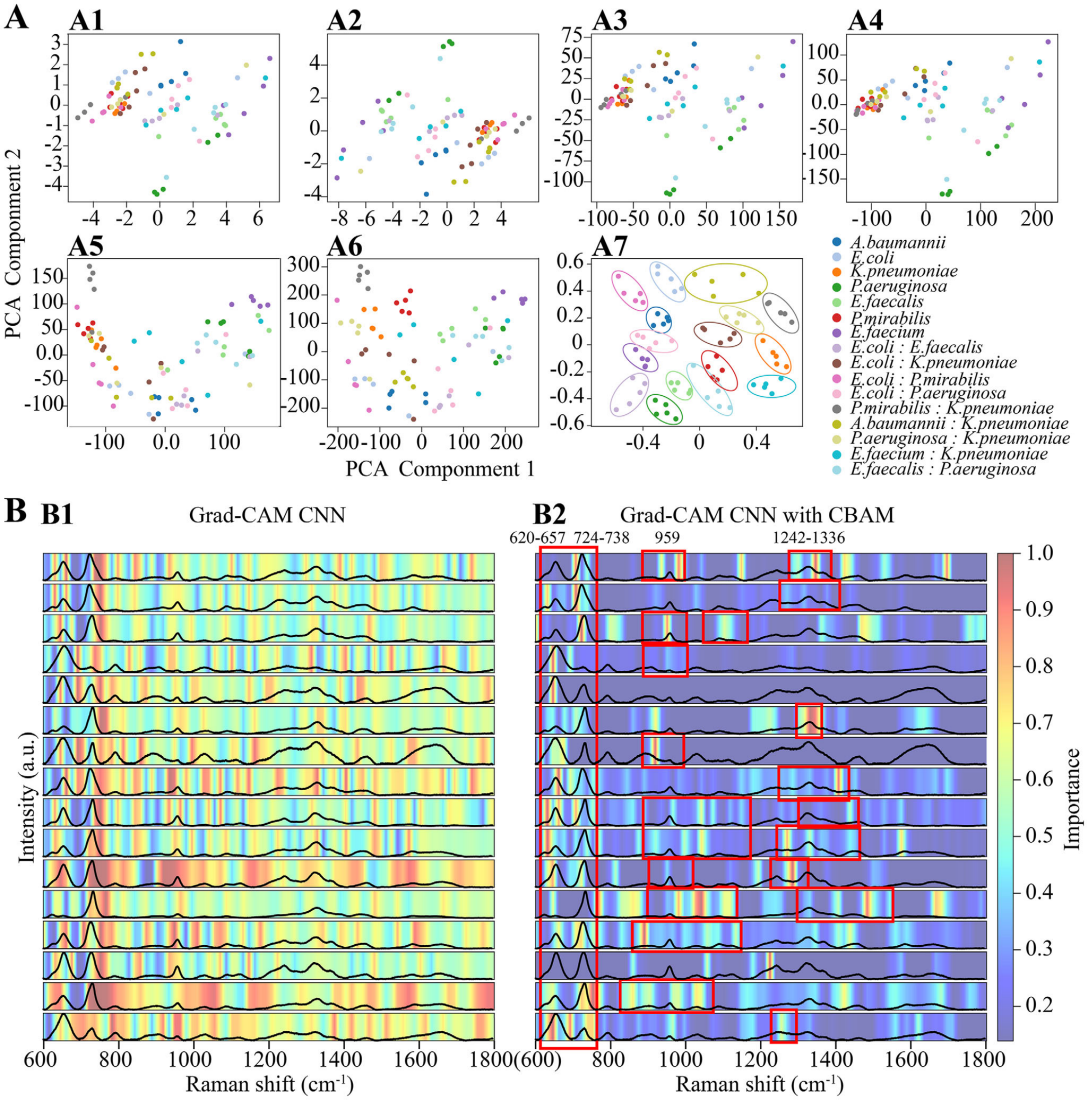
Interpretability analysis of the CNN+CBAM model using PCA projection and Grad‐CAM visualization. A) PCA projections of spectral data at different stages of the CNN+CBAM model pipeline. A1: raw input spectra; A2–A6: feature representations after successive convolutional layers; A7: final output after the CBAM module. B) Grad‐CAM heatmaps comparing the spectral attention of the standard CNN model (B1) and the CNN+CBAM model (B2), each row represents one test sample with the corresponding Raman spectrum overlaid.

To investigate how the CNN+CBAM model processes and transforms spectral data, Principal Component Analysis (PCA) was applied to both the raw inputs and intermediate feature outputs. As shown in **Figure** **3**A1, the original spectra exhibited substantial overlap among bacterial species. With the progression through successive CNN layers (Figure 3A2–A6), increasingly distinct clustering emerged, indicating enhanced class separability. After the CBAM module (Figure 3A7), further improvements in class discrimination were observed, confirming the effectiveness of attention mechanisms in refining spectral representations.

To further interpret model attention, Grad‐CAM was used to generate class‐specific spectral heatmaps (Figure 3B). Compared to the baseline CNN (Figure 3B1), which exhibited dispersed and less specific activation, the CBAM‐enhanced model (Figure 3B2) demonstrated more focused attention aligned with known biochemical markers. In particular, two conserved regions—655 and 725–732 cm^−1^—consistently appeared across multiple species. The 655 cm^−1^ peak arises from C─S and C─C vibrations,^[^ ^34^ ^]^ often found in cysteine/methionine‐rich domains of virulence proteins (e.g., Esp,^[^ ^42^ ^]^ SagA,^[^ ^43^ ^]^ and LasB^[^ ^44^ ^]^). The 725–732 cm^−1^ region represents a biochemical hotspot associated with nucleic acid and amino acid metabolism.^[^ ^34^ ^]^ It primarily arises from adenine ring breathing vibrations—present in key nucleotides like ATP, NADH, and rRNA—alongside C─C and CH_2_ vibrations from lipopolysaccharides (LPS), a hallmark of Gram‐negative bacterial membranes.^[^ ^45^ ^]^ Together, these two spectral regions enable the model to robustly distinguish bacterial phenotypes by capturing essential virulence‐related protein structures and nucleic acid/lipid metabolic activity, offering both biochemical specificity and diagnostic value in pathogen classification.

In addition to the widely recognized peaks at 655 cm and 725 cm^−1^, species‐specific Raman signatures provide further discriminatory power and biological insight into the structural and metabolic characteristics of individual bacteria. The peak at 959 cm^−1^, corresponding to C─N stretching,^[^ ^39^ ^]^ was notably activated in *A. baumannii*, *P. aeruginosa*, and *K. pneumoniae*, likely reflecting active nucleic acid or protein biosynthesis. The 1242–1366 cm^−1^ broad band, associated with amide III and CH bending vibrations,^[^ ^46^ ^]^ was predominantly observed in *E. coli*, indicative of enhanced protein and RNA metabolic activity. Peaks ≈1328–1331 cm^−1^, arising from ═CH in‐plane bending and indole ring breathing (tryptophan),^[^ ^38^ ^]^ were strongly expressed in *A. baumannii* and *P. mirabili*s, pointing to lipid membrane components or motility‐associated proteins. The 895 cm^−1^ peak, attributed to phosphodiester bond and deoxyribose ring vibrations,^[^ ^47^ ^]^ was specifically enhanced in *E. faecium*, possibly linked to glycopeptide‐rich cell wall components in Gram‐positive bacteria. Importantly, in polymicrobial infections, the model demonstrated an ability to integrate and respond to combined spectral features from constituent species. For instance, in mixed cultures of *E. coli* and *K. pneumoniae*, the model showed strong activation at 959, 1090, and 1242–1366 cm^−1^, corresponding to nucleic acid, sugar backbone, and metabolic protein regions. Similarly, *K. pneumoniae* and *P. mirabilis* mixtures exhibited peak activation at 959, 1090, and 1331 cm^−1^.

Complementing the Grad‐CAM results, feature response maps were compared before and after the CBAM module across different infection types (Figure S9, Supporting Information). In the baseline CNN model, feature activation appeared more dispersed, with high sensitivity to spectral noise and limited consistency with known biological marker regions. Upon integrating the CBAM module, the feature representations became more concentrated and biologically meaningful. Across all bacterial sample, two prominent spectral regions ≈655 and 725–732 cm^−1^ were consistently and precisely captured by the CBAM‐refined feature maps. These regions correspond to sulfur‐containing virulence proteins and nucleic acid or tryptophan‐associated signatures, respectively. The accurate localization of these biochemically conserved markers across multiple pathogens suggests that CBAM enables the model to more effectively extract and focus on diagnostically informative spectral features.

Notably, the 959 cm^−1^ region—corresponding to C–N stretching and phosphate backbone vibrations in nucleic acids—exhibited enhanced post‐CBAM activation across nearly all bacterial species, with the exception of *E. faecium*. Its consistent enhancement suggests that the 959 cm^−1^ band serves as a metabolically relevant spectral feature systematically emphasized by the model during bacterial classification. The CBAM output showed improved conformity to the native Raman spectral contours in this region, further emphasizing its biochemical relevance and interpretability. In contrast, the 1242–1366 cm^−1^ region, typically associated with amide III bands, CH_2_ bending, and general protein backbone vibrations, was largely de‐emphasized after CBAM processing, suggesting its limited contribution to class‐specific discrimination. An exception was observed in *K. pneumoniae*, where this region remained prominently activated post‐CBAM—potentially reflecting species‐specific metabolic or membrane‐associated signatures. This selective attenuation across most other species underscores CBAM's capacity to filter non‐discriminative spectral noise and enhance model specificity.

In summary, the integration of Grad‐CAM and CBAM visualization illustrates that the CNN+CBAM model not only improves classification accuracy but also enhances biological interpretability. By emphasizing conserved virulence and metabolic peaks (e.g., 655, 725, and 959 cm^−1^) while suppressing irrelevant signals, the model provides transparent, mechanism‐based insight into Raman‐based bacterial identification—including in complex polymicrobial contexts.

### 2.6 Proportional Estimation of Mixed Bacteria

**Figure 4:**
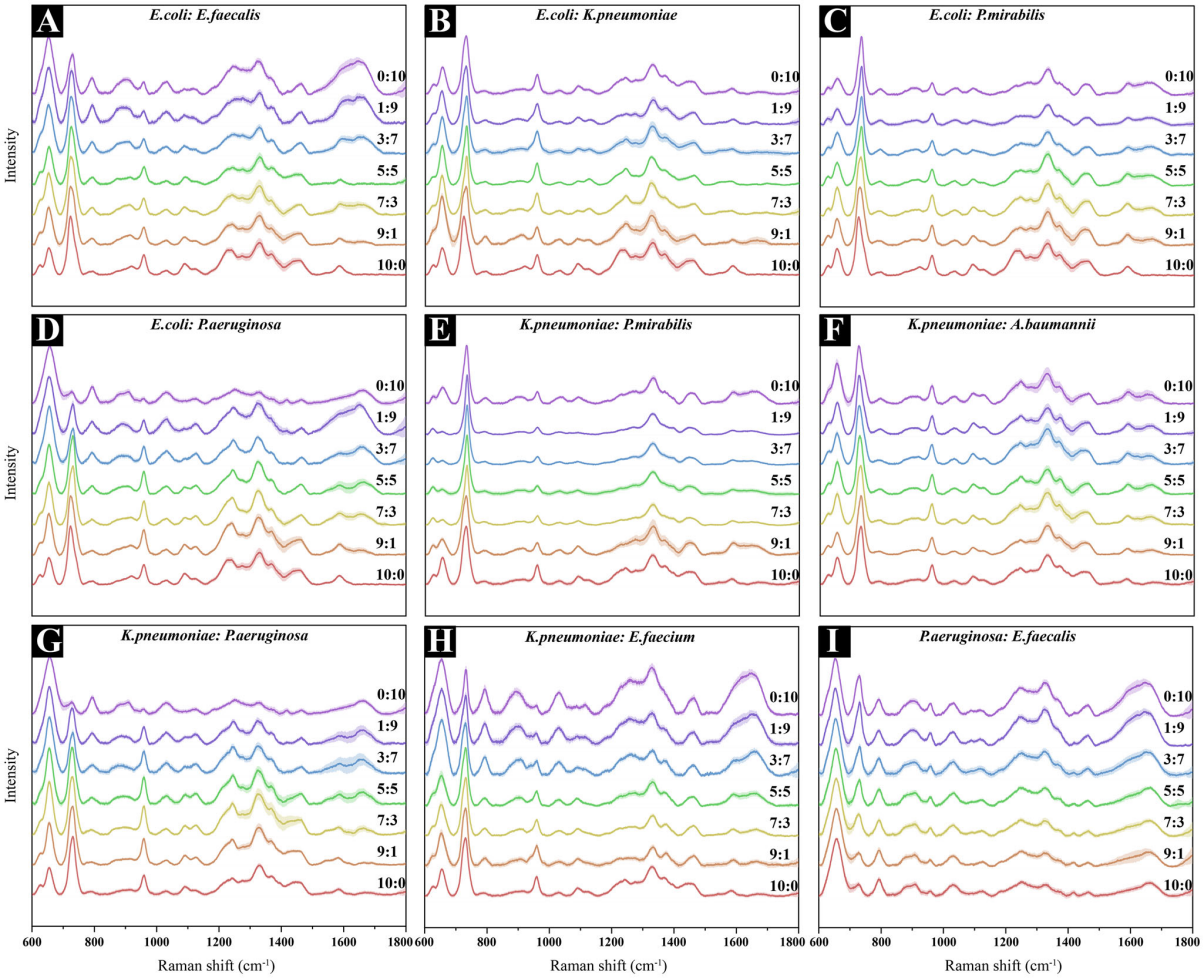
Raman spectra of bacterial mixtures at varying ratios (from 0:10 to 10:0). A) *E. coli*: *E. faecalis*, B) *E. coli*: *K. pneumoniae*, C) *E. coli*: *P. mirabilis*, D) *E. coli*: *P. aeruginosa*, E) *K. pneumoniae*: *P*. *mirabilis*, F) *K. pneumoniae*: *A. baumannii*, G) *K. pneumoniae*: *P. aeruginosa*, H) *K. pneumoniae*: *E. faecium*, and I) *P. aeruginosa*: *E. faecalis*. Each spectral stack shows a gradual shift in intensity and feature composition corresponding to the changing proportions of the two bacterial species.

Beyond identification, quantifying species proportions in polymicrobial infections is clinically vital. SERS spectral data were obtained from mixtures of two bacterial species at various predefined ratios ranging from 0:10 to 10:0. **Figure** **4** illustrates the Raman spectral profiles of different bacterial combinations at varying ratios. As the ratio shifted across the gradient, notable changes in spectral peak intensities were observed, especially for key Raman peaks such as 620–657 and 724–738 cm^−1^, reflecting the compositional changes in the mixtures. These characteristic spectral variations provided a valuable foundation for proportion estimation.

**Figure 5:**
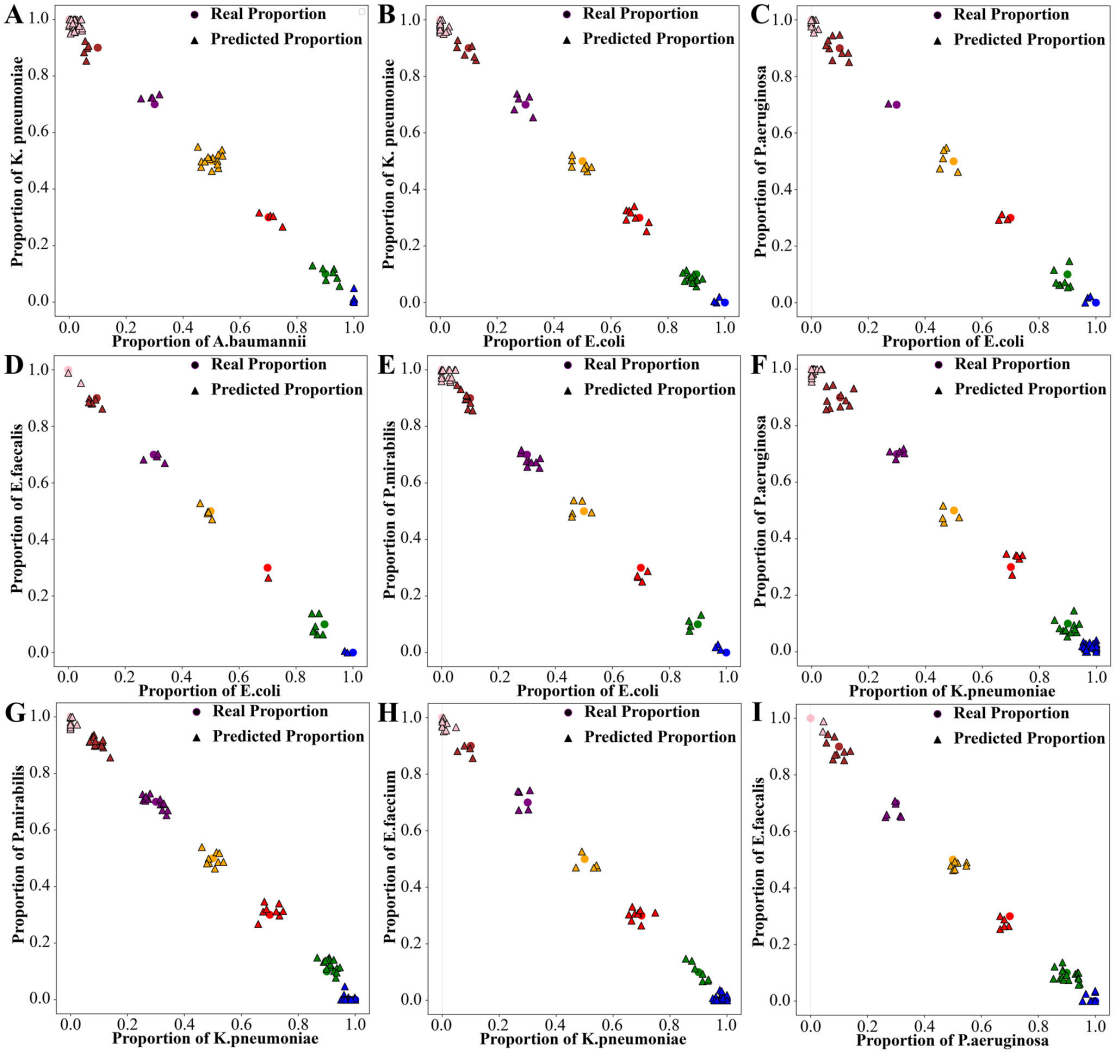
Comparison between real and predicted proportions of bacterial species in binary mixtures. Scatter plots showing the real proportions and predicted proportions of each bacterial species pair in different combinations. A) *A. baumannii*: *K. pneumoniae*, B) *E. coli*: *K. pneumoniae*, C) *P. aeruginosa*: *K. pneumoniae*, D) *E. coli*: *E. faecalis*, E) *E. coli*: *P. mirabilis*, F) *K. pneumoniae*: *P. aeruginosa*, G) *K. pneumoniae*: *P. mirabilis*, H) *K. pneumoniae*: *E. faecium*, I) *P. aeruginosa*: *E. faecalis*. These results demonstrate the model's ability to accurately quantify bacterial composition in mixed samples based on spectral features.

The CNN+CBAM regression model predicted species ratios accurately. As shown in Figure S10A (Supporting Information), the model architecture was similar to that used for classification, except that the output layer was designed for regression tasks. During training, both MAE and loss steadily declined and converged (Figure S10B,C, Supporting Information), indicating effective model optimization. To evaluate the predictive performance, scatter plots were generated for each bacterial combination (**Figure** **5**), each subplot visualizes the actual versus predicted proportions. The model showed high accuracy at extreme ratios, with slight deviations at intermediate ratios that remained within acceptable limits.

Model comparisons (**Table** **2**) showed that CNN+CBAM model achieved the best results, with an MSE of 0.0068, MAE of 0.0392, and an R^2^ of 0.9112, indicating high predictive accuracy and strong fitting capability. In contrast, the standalone CNN model (without CBAM) yielded slightly inferior performance, with an MSE of 0.0163, MAE of 0.0716, and R^2^ of 0.7927. XGBoost performed comparably to CNN, with an MSE of 0.0152, MAE of 0.0644, and R^2^ of 0.7948. The RF model exhibited the lowest performance among the four.

**Table 2:** Comparison of performance indicators of different models in predicting bacterial proportions.

Algorithm	MSE	MAE	R^2^
CNN+CBAM	0.0068	0.0392	0.9112
CNN	0.0163	0.0716	0.7927
XGBoost	0.0152	0.0644	0.7948
RF	0.0278	0.1004	0.6279

CNN: Convolutional Neural Networks; CBAM: Convolutional Block Attention Module; XGBoost: eXtreme Gradient Boosting; RF: Random Forest; MSE: Mean squared error; MAE: Mean absolute error.

Figure S11 (Supporting Information) visualizes actual versus predicted values for each bacterium. Most data points closely aligned with the red reference line, indicating strong consistency between predicted and actual values. Table S2 (Supporting Information) shows species‐specific performance metrics for the CNN+CBAM model. The model performed particularly well in predicting the proportions, exhibiting low errors and high R^2^ values, which suggests high accuracy and robustness across diverse bacterial types. In summary, the CNN+CBAM model demonstrated strong generalization and precision in the task of bacterial proportion prediction, enabling accurate estimation of the relative abundances of different bacterial species in mixed infections.

### 2.7 SERS‐CNN+CBAM Diagnostic Platform for Clinical Samples

**Figure 6:**
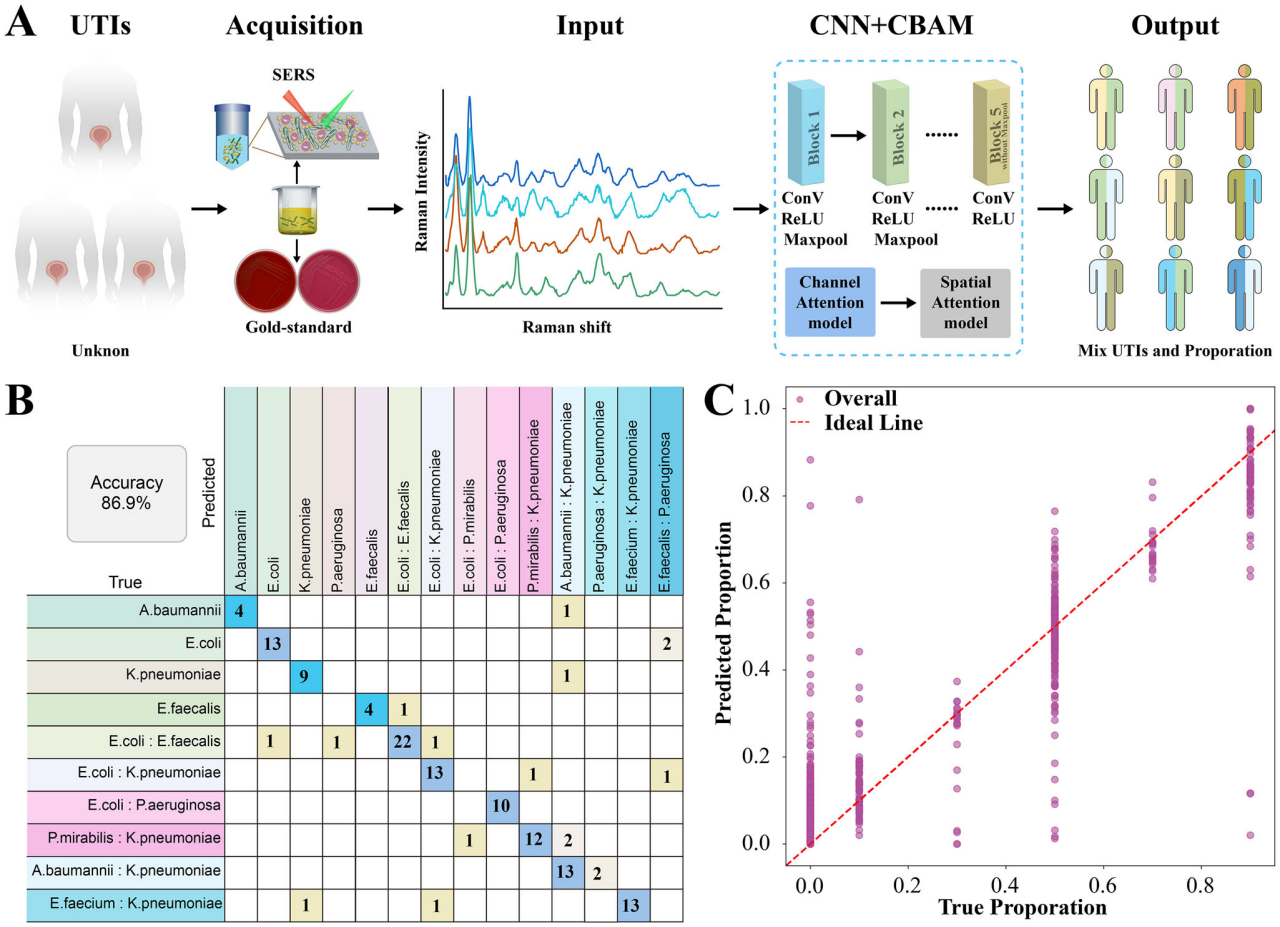
CNN+CBAM model for predicting bacterial composition in urinary tract infections using Raman spectroscopy. A) Schematic of the UTI detection process. B) Confusion matrix showing the classification performance of the CNN+CBAM model with an accuracy of 86.9%. C) Scatter plots comparing predicted and true proportions of individual bacterial species of polymicrobial urinary tract infections with R^2^ = 0.8626.

To validate the clinical applicability of the proposed diagnostic platform, a total of 26 midstream urine samples from patients were collected and analyzed for bacterial identification. Sample preparation was performed as previously described.^[^ ^48^ ^]^ Briefly, 5 mL of urine was filtered and 10‐fold concentrated, then mixed 1:1 with Au@Ag@bPEI substrate for SERS detection (**Figure** **6**A). The classification performance of the CNN+CBAM model is summarized in Figure 6B. The model achieved a high overall classification accuracy of 86.9%, demonstrating strong capability in distinguishing different types of mixed bacterial infections. All mixed infection subtypes achieved individual classification accuracies exceeding 80%. Beyond accuracy, the model yielded a precision of 0.9344, recall of 0.8692, and an F1‐score of 0.8980, indicating not only high predictive precision but also balanced sensitivity across bacterial categories. These metrics underscore the model's suitability for clinical applications, where both specificity and sensitivity are critical.

To validate the model's ability to quantify bacterial proportions, we first prepared synthetic urine samples by spiking *E. coli* and *E. faecalis* at various known ratios. The model accurately predicted the bacterial ratios, with predicted values closely following the diagonal line, indicating strong agreement with the actual proportions (Figure S13, Supporting Information). Quantitative evaluation yielded an MSE of 0.0013, MAE of 0.0224, and R^2^ of 0.7779, demonstrating high accuracy and low error variance in estimating bacterial composition in mixed samples. These results confirm the model's capability to resolve proportional differences in controlled binary mixtures.

Clinical urine samples from nine patients with mixed infections were analyzed to quantify bacterial proportions. Bacterial growth on blood agar and MacConkey agar plates served as the reference for determining the actual composition. The mixed infections included the following cases, with corresponding panels showing bacterial growth on blood agar and MacConkey agar plates: *E. coli*: *E. faecalis*‐9:1 (Figure S14A1,A2, Supporting Information) and 5:5 (Figure S14B1,B2 and C1,C2, Supporting Information); *E. coli*: *K. pneumoniae*‐5:5 (Figure S14D1,D2 and E1,E2, Supporting Information); *A. baumannii*: *K. pneumoniae*‐1:9 (Figure S14F1,F2, Supporting Information) and 3:7 (Figure S14G1,G2, Supporting Information), *E. coli*: *P. aeruginosa*‐9:1 (Figure S14H1,H2, Supporting Information) and 5:5 (Figure S14I1,I2, Supporting Information) (Table S3, Supporting Information). The model demonstrated a high degree of accuracy and robustness in predicting bacterial composition in actual clinical samples, as evidenced by an overall R^2^ of 0.8626 (Figure 6C; Figure S15, Supporting Information), a MSE of 0.0093, and a MAE of 0.0485.

Furthermore, we compared our approach with previous studies on SERS‐based detection of mixed bacteria. Previous studies predominantly utilized gold or silver nanoparticles in conjunction with chemometric techniques or relatively straightforward machine learning models, such as PLS‐DA, PCA‐LDA, and RF. These studies were largely confined to artificial mixtures, food matrices, or blood environments. As detailed in Table S4 (Supporting Information), these approaches generally achieved classification accuracies of ≈87–92% and demonstrated limited efficacy in resolving complex multispecies microbial systems. In contrast, our strategy integrates both substrate and computational innovation. The positively charged Au@Ag@bPEI substrates facilitate enhanced electrostatic interactions with bacterial surfaces, thereby improving signal stability and reproducibility. On the computational front, the CNN‐CBAM framework enables efficient feature extraction and attention‐guided interpretation, which supports reliable identification and quantification in more intricate bacterial mixtures. Our platform successfully discriminated nine clinically relevant bacterial combinations with 95.8% accuracy and R^2^ = 0.9112 under laboratory conditions, surpassing the performance of most prior reports. Importantly, robust performance was also maintained in real clinical urine samples (accuracy 86.9%, R^2^ = 0.8626). These results highlight the innovative and competitive nature of our approach, illustrating its potential for translation into clinical practice for multiplexed pathogen detection.

In summary, this study demonstrates the feasibility of integrating Raman spectroscopy with deep learning to achieve rapid, label‐free identification and quantification of urinary pathogens. Beyond accurate species classification, the incorporation of convolutional and attention mechanisms allowed the model to distinguish subtle spectral differences, even in complex mixed infections. This approach not only shortens the diagnostic timeline compared to traditional culture‐based methods but also opens avenues for real‐time monitoring of pathogen dynamics in clinical and environmental settings. Moreover, the ability to quantify bacterial proportions may support early detection of polymicrobial infections and guide more precise antimicrobial interventions. Combining SERS with AI‐driven models could be extended to broader applications such as antimicrobial resistance profiling, infection source tracing, and integration into portable diagnostic devices for point‐of‐care use.

## 3 Conclusion

In this study, we developed an interpretable, label‐free diagnostic platform that integrates a functionally engineered SERS substrate with an attention‐based deep learning model for the identification and quantification of UTIs. The Au@Ag@bPEI plasmonic substrate, designed with a positively charged surface, enabled effective electrostatic bacterial capture and consistent Raman signal acquisition across diverse samples. Coupling this substrate with a CNN model enhanced by CBAM, we achieved both high classification accuracy (95.8%) and reliable bacterial proportion prediction (R^2^ = 0.9112), significantly outperforming conventional classifiers such as CNN, XGBoost, and RF. The incorporation of attention mechanisms provided not only improved performance but also mechanistic interpretability by localizing Raman spectral features associated with nucleic acids, proteins, and virulence‐related metabolites. Clinical validation using urine samples confirmed the robustness and translational potential of the platform, reaching 86.9% classification accuracy and providing reliable estimates of bacterial ratios with 0.8626. Overall, this study establishes a robust and interpretable SERS–AI framework that resolves the complex spectral signatures characteristic of mixed bacterial populations, thereby facilitating timely and precise therapeutic decision‐making. By capturing both conserved and species‐specific spectral features, the model provides a strong theoretical foundation for SERS‐based spectral deconvolution in complex microbial environments. Its high generalizability and interpretability support broad applicability in point‐of‐care diagnostics, hospital infection control, and environmental microbial surveillance—offering a practical and explainable solution to the growing challenge of mixed bacterial infections.

## 4 Experimental Section

### Chemicals and Materials

Chloroauric acid (HAuCl4·3H_2_O), branched polyethyleneimine (bPEI), and silver nitrate were purchased from Sigma–Aldrich, Inc.(St. Louis, USA); Sodium citrate (C_6_H_5_Na_3_O_7_·2H_2_O), Glutaraldehyde solution (50%), and ascorbic acid were purchased from Macklin Biochemical Co., Ltd.(Shanghai, China); LB solid agar, MacConkey agar medium, and LB agar were purchased from Haibo Biological Technology Co., Ltd.(Qingdao, China); Blood agar plates were purchased from Beckman Coulter, Inc. (Brea, USA); HEPES buffer (1M) was obtained from Biosharp (Hefei, China); Deionized water (18.2 MΩ) was used in all experiments.

### Mixed UTIs Identified and Bacterial Culture

A retrospective analysis of urine culture results from patients at the Second Hospital of Tianjin Medical University over the past two years was conducted to identify the predominant pathogens associated with mixed UTIs. For inclusion, bacterial species involved in mixed infections were included only if they had been isolated in at least 10 documented cases.

For model training and validation, a total of 35 bacterial strains were selected in this study, comprising five strains each of *E. coli*, *A. baumannii*, *E. faecium*, *E. faecalis*, *K. pneumoniae*, *P. aeruginosa*, and *P. mirabilis*. All strains were isolated from UTIs at the Second Hospital of Tianjin Medical University and identified using matrix‐assisted laser desorption/ionization time‐of‐flight mass spectrometry (MALDI‐TOF MS). The collected isolates were cultured in 100 mL of inactivated LB medium at 37 °C with shaking at 180 rpm for overnight. Portions of the resulting bacterial suspensions were stored at −80 °C until further analysis.

### Synthesis of Au@Ag@bPEI NPs and Characterization

The synthesis method of Au NPs adopted the traditional Turkevich method.^[^ ^49^ ^]^ Au@Ag core–shell NPs were subsequently prepared via a seed‐mediated growth approach, in which the outer surface was modified with bPEI to alter the surface charge distribution. Detailed synthesis and characterization protocols are provided in Supporting Information.

### Sample Preparation and Raman Spectroscopy Measurements

For model establishment, two bacterial strains were independently cultured until reaching the logarithmic growth phase, quantified by plate counting, and then adjusted to a concentration of 10^6^ CFU mL^−1^. The two strains were then mixed in varying volume ratios of 10:0, 9:1, 7:3, 5:5, 3:7, 1:9, and 0:10 to create a series of mixed samples with defined concentration gradients. Subsequently, 1 mL of each mixed sample was washed with deionized water and concentrated 10‐fold prior to SERS analysis.

For spectral acquisition, 20 µL of the concentrated Au@Ag@bPEI NPs was mixed with 20 µL of the prepared bacterial suspension and incubated at room temperature for 10 min. A 5 µL aliquot of the mixture was then drop‐cast onto a silicon slide and air‐dried for analysis. SERS spectra were acquired using a portable Raman spectrometer (Zolix FI‐FO785S‐Plus, China) equipped with a 785 nm laser, a resolution of 3.5 cm^−1^, laser power of 350 mW, and a spot size of 100 µm. Spectral acquisition was performed over the 600–1800 cm^−1^ range with five accumulations. To ensure data quality and reproducibility, at least 20 spectra were collected for each sample.

To clinically evaluate the model's performance, midstream urine samples from 26 patients with UTIs were collected and used for bacterial species identification. The trained model was applied to identify bacterial species directly from these clinical specimens. Furthermore, nine additional mixed‐infection urine samples were analyzed to evaluate the model's capability in predicting bacterial composition ratios.

All bacterial cultures and mixture preparations were performed in independent batches on separate days to ensure reproducibility. Clinical urine samples were collected from different patients at different time points to validate the robustness of the model in real clinical settings. Detailed experimental procedures are provided in Supporting Information.

### Raman Data Processing and Machine Learning

Each individual SERS spectrum was preprocessed using LabSpec6 software (Horiba JY). A linear baseline correction was first applied to eliminate background interference, followed by min–max normalization to scale the spectral intensities within a standardized range.

To identify and quantify mixed bacterial populations based on SERS data, several machine learning algorithms were employed in this study, including two widely used classical methods‐XGBoost and RF‐as well as a deep learning model based on a CNN, and an advanced architecture combining CNN with a CBAM. For all models (CNN, CNN+CBAM, XGBoost, and RF), the same preprocessed SERS spectra were used as input.

Prior to model training, the dataset was randomly split into training, testing, and validation subsets at a ratio of 60:20:20. The testing set, which remained entirely independent of the training process, was used exclusively for performance evaluation of each trained model. To assess model robustness and generalizability, fivefold cross‐validation was performed using the cv function with the number of folds set to 5. The detailed architecture and parameter settings of the CNN+CBAM model are provided in the Supplementary Information.

### Interpretation Methods for CNN‐CBAM Classifiers

To better interpret the CNN+CBAM model, three complementary techniques were employed. First, PCA^[^ ^50^ ^]^ was applied to both raw Raman spectra and CNN‐CBAM extracted. Second, Grad‐CAM^[^ ^51^ ^]^ was used to identify regions within the spectra that contributed most to classification decisions. Third, to interpret the attention weights within the CBAM module, attention weights from the CBAM module^[^ ^25^ ^]^ were extracted. For each class, one representative sample was selected, and its original spectrum, convolutional features, and CBAM‐weighted output were plotted.

### Ethics Approval and Consent to Participate

This study was approved by the Ethics Committee of The Second Hospital of Tianjin Medical University (No. KY2024K004).

## Conflict of Interest

The authors declare no conflict of interest.

## Supporting information



Supporting Information

## Data Availability

The data that support the findings of this study are available from the corresponding author upon reasonable request.
