# Supplementary material for: An Interpretable SERS–AI Platform for Rapid and Quantitative Diagnosis of Polymicrobial UTIs: Powered by Positively Charged Plasmonic Nanoparticles and Attention‐Based Deep Learning
Source: Adv Sci (Weinh). 2025 Sep 24;12(46):e13502. doi: 10.1002/advs.202513502 (PMC12697763; doi:10.1002/advs.202513502)
Supplement: Supplementary file 1 — Supporting Information [file ADVS-12-e13502-s001.doc]

**Supplementary Information**

**An Interpretable SERS–AI Platform for Rapid and Quantitative Diagnosis of Polymicrobial UTIs: Powered by Positively Charged Plasmonic Nanoparticles and Attention-Based Deep Learning**

Zhonghua Shen^1#^, Linguo Xie^2#^, Yuwei Hou^3#^, Junjie Liang^2^, Yuchi Jia^2^, Haipeng Zhang^2^, Zhenli Sun^4^, Jingjing Du^5^, Zeying He^1*^, Chunyu Liu^2*^, Wenjing Liu^1*^

1 Key Laboratory for Environmental Factors Control of Agro-product Quality Safety, Agro-Environmental Protection Institute, Ministry of Agriculture and Rural Affairs, Tianjin 300191, China

2 Department of Urology, Tianjin Institute of Urology, The Second Hospital of Tianjin Medical University, Tianjin, 300211, China

3 Department of Radiology, Tianjin Beichen Hospital, Tianjin, 300400, China

4 MOE Key Laboratory of Resources and Environmental System Optimization, College of Environmental Science and Engineering, North China Electric Power University, Beijing 102206, China

5 State Key Laboratory of Environmental Chemistry and Ecotoxicology, Research Center for Eco-Environmental Sciences, Chinese Academy of Sciences, Beijing 100085, China

# Equal contributors

*Corresponding Authors: Corresponding Authors：Zeying He [(hezeying@caas.cn);](mailto:(hezeying@caas.cn);) Chunyu Liu (liuchunyu@tmu.edu.cn), Wenjing Liu (liuwenjing@caas.cn)

**Contents**

1. Experimental Section
2. Figure S1. Distribution of mixed bacterial infections in urinary tract infection (UTI) cases
3. Figure S2. X-ray photoelectron spectroscopy (XPS) spectra of the Au@Ag@bPEI nanocomposite
4. Figure S3. Raman spectra of R6G detected by Au@Ag@bPEI nanoparticles at varying concentrations
5. Figure S4. SEM images of *E. coli* and *E. faecalis* associated with Au@Ag@bPEI nanoparticles
6. Figure S5. SERS characterization of *E. coli* and *E. faecalis* using Au@Ag@bPEI nanoparticles.
7. Figure S6. **Detection of** E. coli **using Au@Ag@bPEI nanoparticles via SERS**
8. Table S1：Raman peak assignments of single and mixed bacteria
9. Figure S7. Accuracy and loss curves of the CNN+CBAM model across four cross-validation folds
10. Figure S8. Radar plot of classification performance metrics for 16 bacterial categories
11. Figure S9. Visualization of feature responses before and after CBAM enhancement for representative bacterial species and mixed infections
12. Figure S10. Architecture and performance of the CNN+CBAM model for bacterial mixture ratio prediction using Raman spectra
13. **Figure. S11.** **Scatter plots comparing predicted and true proportions of individual bacterial species in binary mixtures**
14. Table S2：Performance indicators of different bacterial species in proportion prediction in the CNN+CBAM model
15. Figure S12. SERS spectra of urine samples spiked with *E. faecalis* and *E. coli* at varying concentrations, using Au@Ag@bPEI core-shell substrates
16. Figure S13. Representative images of urinary samples with synthetic binary mixtures *E. coli* and *E. faecalis* at different ratios
17. Figure S14. Bacterial culture results from urine samples of nine patients with mixed infections
18. Table S3: Bacterial quantification in nine patients with mixed infections
19. Figure S15. Comparison between real and predicted proportions of bacterial species of mixtures in urine
20. Table S4: Comparative overview of SERS substrates, modeling approaches, and their performance for mixed bacterial species analysis

**Experimental Section**

**Synthesis of Au@Ag@bPEI NPs**

The synthesis method of Au nanoparticles (NPs) adopted the traditional Turkevich method. Specifically, 1 mL of 1% (w/v) chloroauric acid (HAuCl₄·3H₂O) solution was added to 100 mL of ultrapure water in a round-bottom flask and heated to boiling under vigorous stirring. Once the solution reached a rolling boil, 1 mL of 1% (w/v) sodium citrate solution was rapidly added. The mixture was kept boiling for approximately 15 minutes, during which the color changed from pale yellow to deep red, indicating the successful formation of Au NPs. The resulting colloidal solution was cooled to room temperature and stored at 4 °C for further use.

Au@Ag NPs were synthesized using a seed-mediated growth method, 3 mL of Au NPs in a 15 mL centrifuge tube was shaken at 25°C, 300 rpm. 125 μL of 10 mmol/L AgNO₃ was added slowly to coat Au seeds with Ag⁺. Then, 125 μL of 10 mmol/L ascorbic acid was added. The mixture was shaken for 20 more minutes to fully form the core-shell structure.

Au@Ag NPs outer surface were modified by bPEI to alter the surface charge distribution. The obtained Au@Ag NPs were washed twice with ultrapure water and dissolved in ultrapure water. A 1 mg/mL bPEI solution was added in a 1:1 volume ratio, followed by ultrasonic treatment for 30 minutes. The mixture was then shaken at 300 rpm for 2 hours at 25°C. The bPEI-modified Au@Ag@bPEI NPs were obtained by centrifugation. The obtained Au@Ag@bPEI NPs were concentrated by centrifugation at 12,000 rpm for 10 minutes at 4℃, resulting in a 10-fold concentration before use.

**Characterization**

The synthesized Au@Ag@bPEI nanoparticles were deposited onto copper grids and imaged using a high-resolution transmission electron microscope (HRTEM; FEI-Talos F200S) operated at 200 kV. The crystal structure was determined by capturing selected area electron diffraction (SAED) patterns from the HRTEM images. Further physicochemical characterization was performed using Zeta potential analysis (ZetaPALS; Brookhaven Instruments) and X-ray photoelectron spectroscopy (XPS; Nexsa; Thermo Fisher). Zeta potential measurements were conducted to evaluate the surface charge of the nanoparticles. Standard bacterial strains, *E. coli* ATCC 25922 were purchased from the BioTeke Corporation Co.,Ltd. (Wuxi, China) and used to evaluate the detection capability of the synthesized Au@Ag@bPEI NPs.

**Sample preparation for SEM Characterization**

Bacterial isolates were obtained from 5 mL of patient urine co-infected with *E. coli* and *E. faecalis*. After passing through a 5 µm filter to remove host cells, bacteria were collected by centrifugation at 8,000 rpm for 5 min. The bacterial pellet was subsequently washed twice with 5 mM HEPES buffer, and resuspended in 100 µL of the same buffer. The suspension colloid was mixed 1:1 (v/v) with Au@Ag@bPEI and incubated at ambient temperature for 10 min. A 5 µL aliquot was then deposited onto silicon substrates and allowed to adhere for 10 min. The samples were fixed with 2.5% glutaraldehyde for 120 min, washed three times with HEPES buffer, dehydrated through a graded ethanol series (30-100%), and subjected to critical point drying. Finally, the samples were mounted on SEM stubs using conductive tape for imaging.

**Clinical Validation**

In this study, midstream urine samples were collected from patients presenting to the department of urology with suspected UTIs. A total of 10 mL urine was obtained from each patient, with 5 mL subjected to conventional urine culture and the remaining 5 mL used for Raman spectroscopy analysis. All processing steps were performed under aseptic conditions. Initially, host cells were depleted using a 5 µm membrane filter. The filtrate was then subjected to two successive centrifugation washes (8,000 rpm, 5 minutes each) using 1 mL of deionized water per wash. Finally, the bacterial pellet was resuspended in 100 ul of deionized water for subsequent Raman spectroscopic analysis.

To assess potential matrix effects from urine components, both control and spiked samples were systematically prepared. Sterile, filter-sterilized midstream urine was used either alone (blank control) or spiked with *E. faecalis* or *E. coli* suspensions at graded concentrations (10^4^-10^7^ CFU/mL). Pure bacterial suspensions at 10^7^ CFU/mL concentrations served as additional references. SERS spectra were then collected from these parallel groups to directly compare spectral features between pure cultures and urine-based samples across different bacterial loads.

For bacterial identification, a total of 26 clinical urine samples were analyzed, which included both single-species and multiple polymicrobial combinations. The samples included: 1 sample of *A. baumannii*, 3 samples of *E. coli*, 2 samples of *K. pneumoniae*, 1 sample of *E. faecalis*, 5 samples of *E. coli* + *E. faecalis*, 3 samples of *E. coli* + *K. pneumoniae*, 2 samples of *E. coli* + *P. aeruginosa*, 3 samples of *P. mirabilis* + *K. pneumoniae, 3 samples of A. baumannii + K. pneumonia*e, and 3 samples of *E. faecalis* + *K. pneumoniae*.

A controlled simulation experiment was performed to evaluate the capability of Raman spectroscopy in quantifying bacterial proportion. *E. coli* and *E. faecalis* were cultured to the logarithmic growth phase, quantified by plate counting, and subsequently harvested by centrifugation at 8,000 rpm for 5 min. The bacterial cells were washed twice with sterile deionized water, and adjusted to a final concentration of 1×10^6^ CFU/mL. Defined ratios of the two bacterial species (10:0, 9:1, 7:3, 5:5, 3:7, 1:9, 0:10) were prepared, centrifuged, and resuspended in sterile, filter-sterilized urine to yield mixtures with a total bacterial load of 1×10^6^ CFU/mL. Subsequent steps followed the clinical sample preparation protocol, and five Raman spectra were collected for each infection-positive mixture.

Simultaneously, the prediction of bacterial proportions was conducted on nine clinical urine samples exhibiting mixed infections: three samples with *E. coli* + *E. faecalis*; two samples with *E. coli* + *K. pneumonia*e; two samples with *A. baumannii* + *K. pneumoniae*, and two samples with *E. coli* + *P. aeruginosa*. For each sample, 5 µL of urine was plated on blood agar and MacConkey agar plates and incubated overnight. Colony counts were then used to determine the composition and calculate the bacterial ratios.

**Structure of CNN and CBAM**

The structure of CNN and CBAM was constructed to analyze Raman spectral data for classification and identification of the proportion of mixed bacteria. The CNN module consists of five convolutional blocks. Each convolutional block consists of a convolutional layer (Conv) and a ReLU activation function, and all blocks except the fifth include a max-pooling layer (MaxPool).

The Conv extracted local features by sliding the convolution kernel, such as vibration patterns and chemical information from the spectra. The number of convolution kernels started from 16 and gradually increases to 256 layer by layer, capturing higher-level features step by step. The ReLU activation function introduced non-linear transformation, enhancing the model's ability to represent complex spectral patterns. The max-pooling layer (MaxPool) reduced the feature dimension while retaining the main features.

After the last Conv block, CBAM was introduced to enhance the model's ability to focus on key spectral features. CBAM includes the following two parts: Channel attention module, which weighted the importance of different channels, highlighting the feature channels related to specific chemical information or molecular vibration patterns; Spatial attention module, By calculating the attention weights in the spatial dimension, it emphasized the important band positions in the spectra, thereby enhancing the sensitivity to key regions.

CBAM combined the attention mechanisms in both the channel and spatial aspects, enabling the model to better capture the important information in the spectral data. Based on the features extracted by the convolutional and attention modules, a flattening operation was performed to convert the three-dimensional feature map into a one-dimensional vector. Subsequently, the feature vector passes through two fully-connected layers with 128 and 64 neurons respectively, and was finally used for classification and regression output.

For classification, the model was trained using the Adam optimizer with a learning rate of 0.001. The categorical cross-entropy loss function was used, and model performance was evaluated based on accuracy, precision, recall, and F1-score. For regression, the model was trained using the Adam optimizer with a learning rate of 0.001. The training objective was to minimize the mean squared error (MSE), and performance was further evaluated using mean absolute error (MAE) and the coefficient of determination (R²). Training was conducted for 50 epochs with a batch size of 32. Five-fold cross-validation was used to identify the best model, which was then evaluated on an independent test set to assess generalization ability.

In this study, The MATLAB R2023a and Python 3.9 were used to finish machine learning.


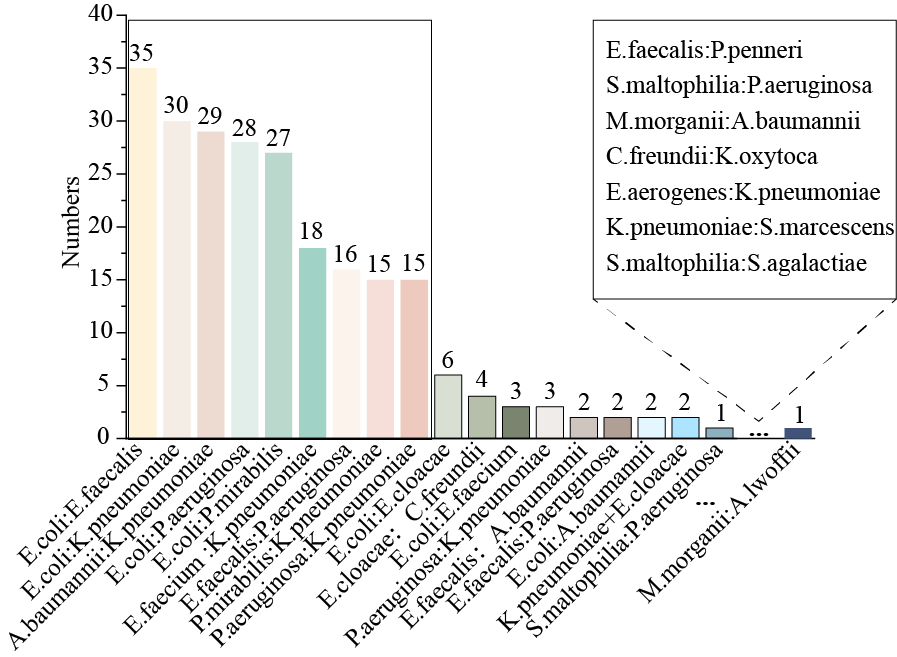


**Figure S1.** **Distribution of mixed bacterial infections in urinary tract infection (UTI) cases.**


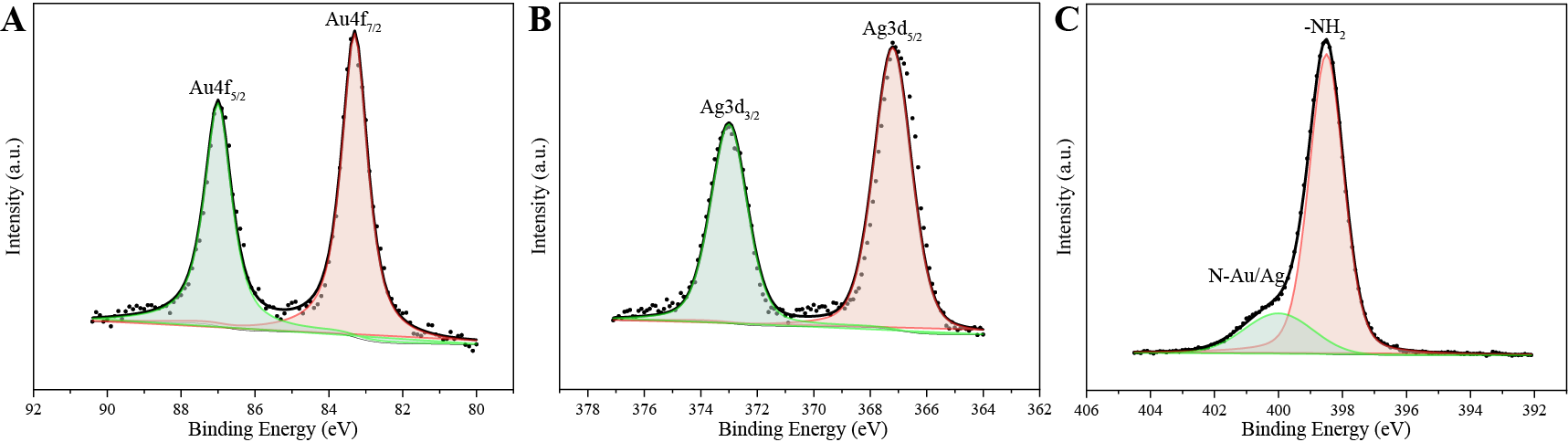


**Figure S2. X-ray photoelectron spectroscopy (XPS) spectra of the Au@Ag@bPEI nanocomposite.** **(A)** High-resolution XPS spectrum of Au showing two characteristic peaks at approximately 83.3 eV and 87.0 eV corresponding to Au 4f₇/₂ and Au 4f₅/₂. **(B)** High-resolution XPS spectrum of Ag displaying peaks at 367.2 eV (Ag 3d₅/₂) and 373.0 eV (Ag 3d₃/₂). **(C)** XPS spectrum of N 1s revealing a strong peak at 398.5 eV attributed to amino groups (-NH₂) from bPEI, and a secondary peak at 400.0eV assigned to N-Au/Ag coordination.


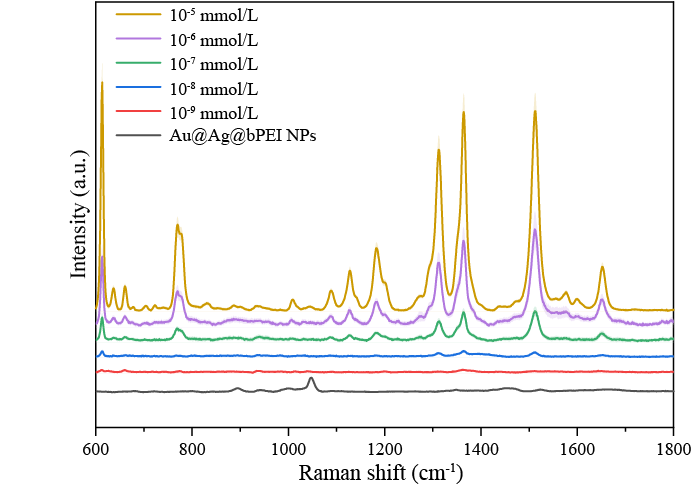


**Figure S3. Raman spectra of R6G detected by Au@Ag@bPEI nanoparticles at varying concentrations.**


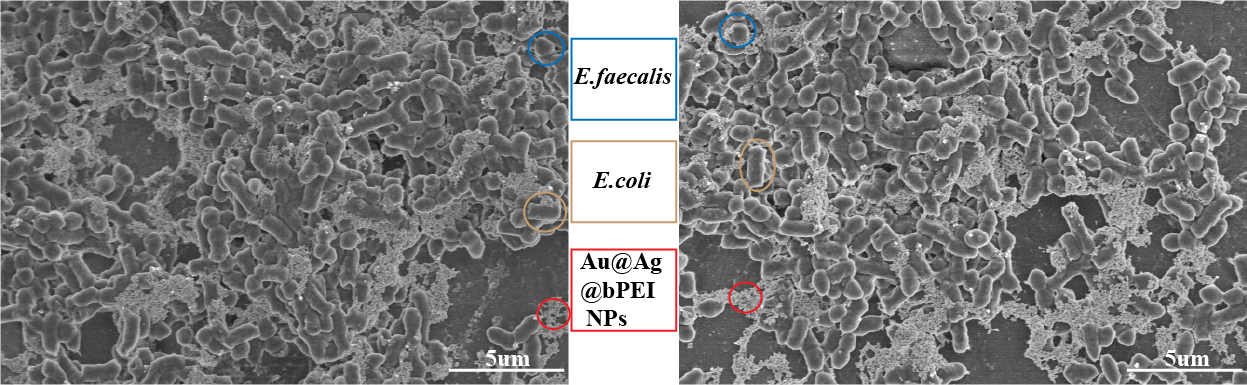


**Figure S4. SEM images of *E. coli* and *E. faecalis* associated with Au@Ag@bPEI nanoparticles.**

SEM images (Figure S4) show Au@Ag@bPEI nanoparticles associated with bacterial surfaces, confirming successful formation of the composite structure on the silicon wafer.


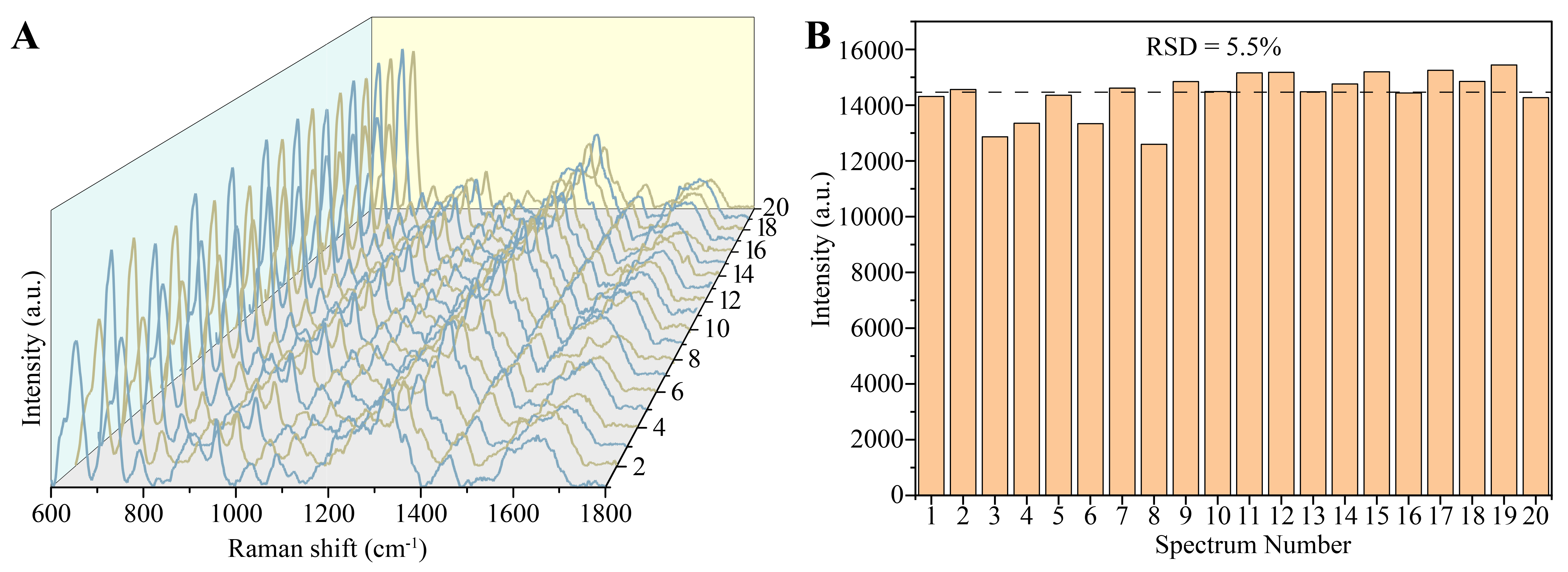


****Figure S5.** **SERS characterization of *E. coli* and *E. faecalis* using Au@Ag@bPEI nanoparticles.** (A)** Twenty SERS spectra of *E. coli* with *E. faecalis* were collected from randomly selected locations. **(B)** Intensity of the characteristic peak at 730 cm^-1^ for *E. coli* with *E.faecalis*, showing a relative standard deviation (RSD) of 5.5%.

The SERS spectra of *E. coli* mixed with *E. faecalis*, obtained from 20 randomly selected spots, exhibited consistent characteristic peaks (Figure S5A), with the peak at 730 cm⁻¹ showing a relative standard deviation (RSD) of 5.5% (Figure S5B), indicating reliable signal reproducibility. Such an RSD value is well within the commonly accepted range for reproducible SERS measurements, with previous studies reporting batch-to-batch variations of 5-10% in colloids^[1-3]^, thereby demonstrating excellent signal reproducibility.

**
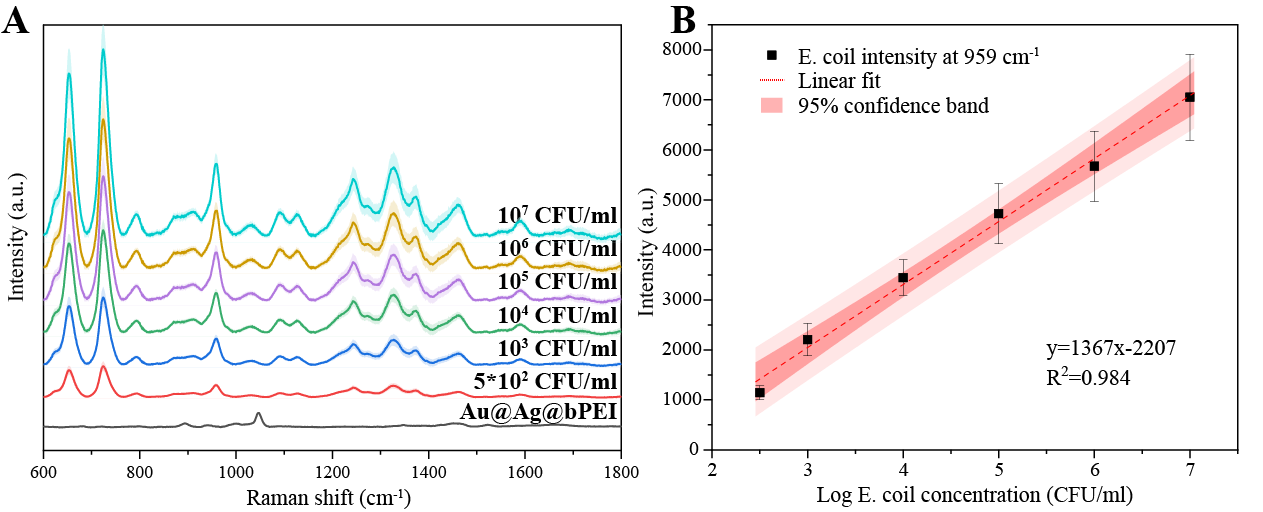
**

****Figure S6.** **Detection of** E. coli **using Au@Ag@bPEI nanoparticles via SERS.** (A)** SERS spectra of E. coli at different concentrations ranging from 5×10² to 10⁷ CFU/mL using Au@Ag@bPEI nanoparticles as the substrate. **(B)**  Calibration curve of Raman intensity at 959 cm⁻¹ versus the logarithm of E. coli concentration, showing a strong linear correlation (R² = 0.984).

| **Table S1：**Raman peak assignments of single and mixed bacteria | |
| --- | --- |
| Frequency （cm^-1^） | Assignments |
| 620-640 | C-C tw (G, Phe) or C-N str (Tyr)^[4]^ |
| 644-657 | C−S str, C−C tw G (C−S) (proteins, nucleic acids)^[5]^ |
| 724−738 | ρ(CH2) adenine, trans conformation of (C-S), or tryptophan^[5]^ |
| 788 | C5’-O-P-O-C3’ phosphodiester bands in DNA^[6]^ |
| 794 | C-S guanine (nucleic acids)^[7]^ |
| 885 | Disaccharide (cellobiose), (C-O-C) skeletal mode^[8]^ |
| 895 | Phosphodiester, Deoxyribose^[9]^ |
| 898 | Monosaccharides (β-glucose), (C-O-C) skeletal mode^[8]^ |
| 904 | C-C skeletal stretching^[10]^ |
| 918 | Proline, hydroxyproline^[11]^ |
| 959 | C-N stretching vibration^[12]^ |
| 1030 | ν(CC) skeletal, keratin (protein assignment)^[13]^ |
| 1032 | Phenylalanine^[11]^；Proline^[14]^ |
| 1037 | C-O-C stretching vibrations of the saccharide^[15]^ |
| 1090 | nucleic acids^[16]^ |
| 1116 | CH2,6 in-plane bend and C1-Cα-Hα bend^[17]^ |
| 1124 | Carbohydrates, C-N and C-C stretching, polysaccharide^[12]^ |
| 1234 | A concerted ring mode^[18]^ |
| 1242 | Amide III (β sheet and random coils)^[19]^ |
| 1252 | Guanine, cytosine (NH2)^[20]^ |
| 1275 | Amide III^[8]^ |
| 1325-1336 | =CH in plane(lipid) or amide III (protein)^[21]^ |
| 1367 | υs (CH3) (phosphplipids)^[19]^ |
| 1415 | nucleic acids^[22]^ |
| 1445 | δ(CH2), δ(CH3), (protein)^[13]^ |
| 1452-1466 | CH2 def^[4]^ |
| 1586 | C=C (lipid)^[23]^ |
| 1643 | Amide I^[24]^ |
| 1653 | Lipid (C=C stretch)^[25]^ |
| 1662 | Nucleic acid modes^[26]^ |
| ρ: rocking vibration; υ: stretching vibration; υs: symmetric stretching vibration; δ: bending vibration | |


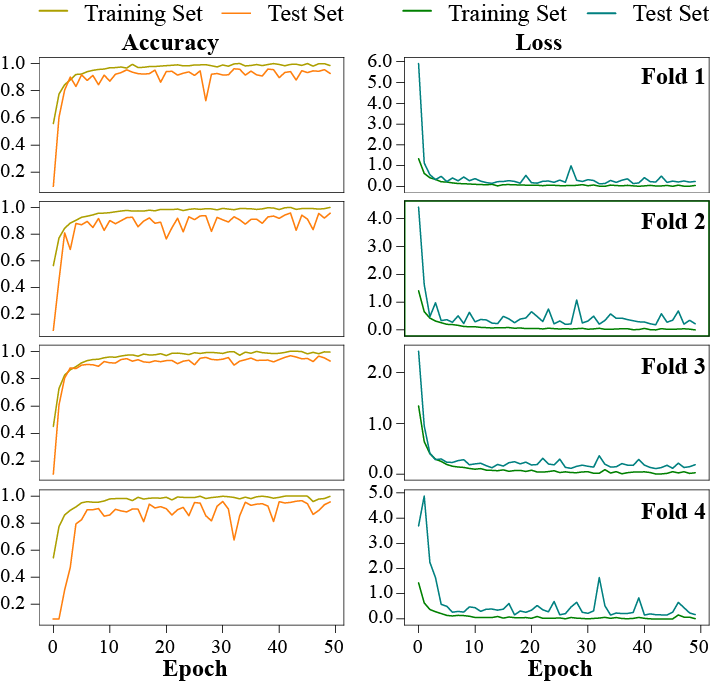


**Figure S7. Accuracy and loss curves of the CNN+CBAM model across four cross-validation folds.** Training and test set accuracies (left) and loss values (right) are shown over 50 training epochs for each fold in five cross-validation.


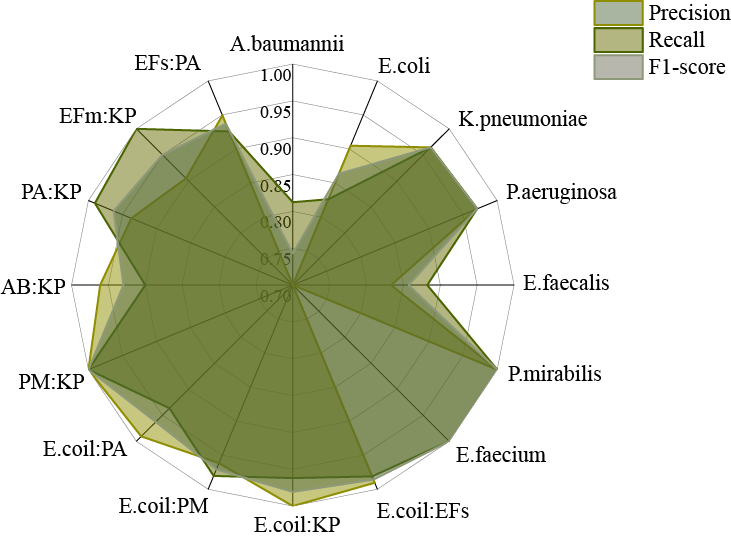


**Figure S8. Radar plot of classification performance metrics for 16 bacterial categories.** Radar chart illustrating the precision, recall, and F1-score of the trained model in classifying 16 bacterial categories.


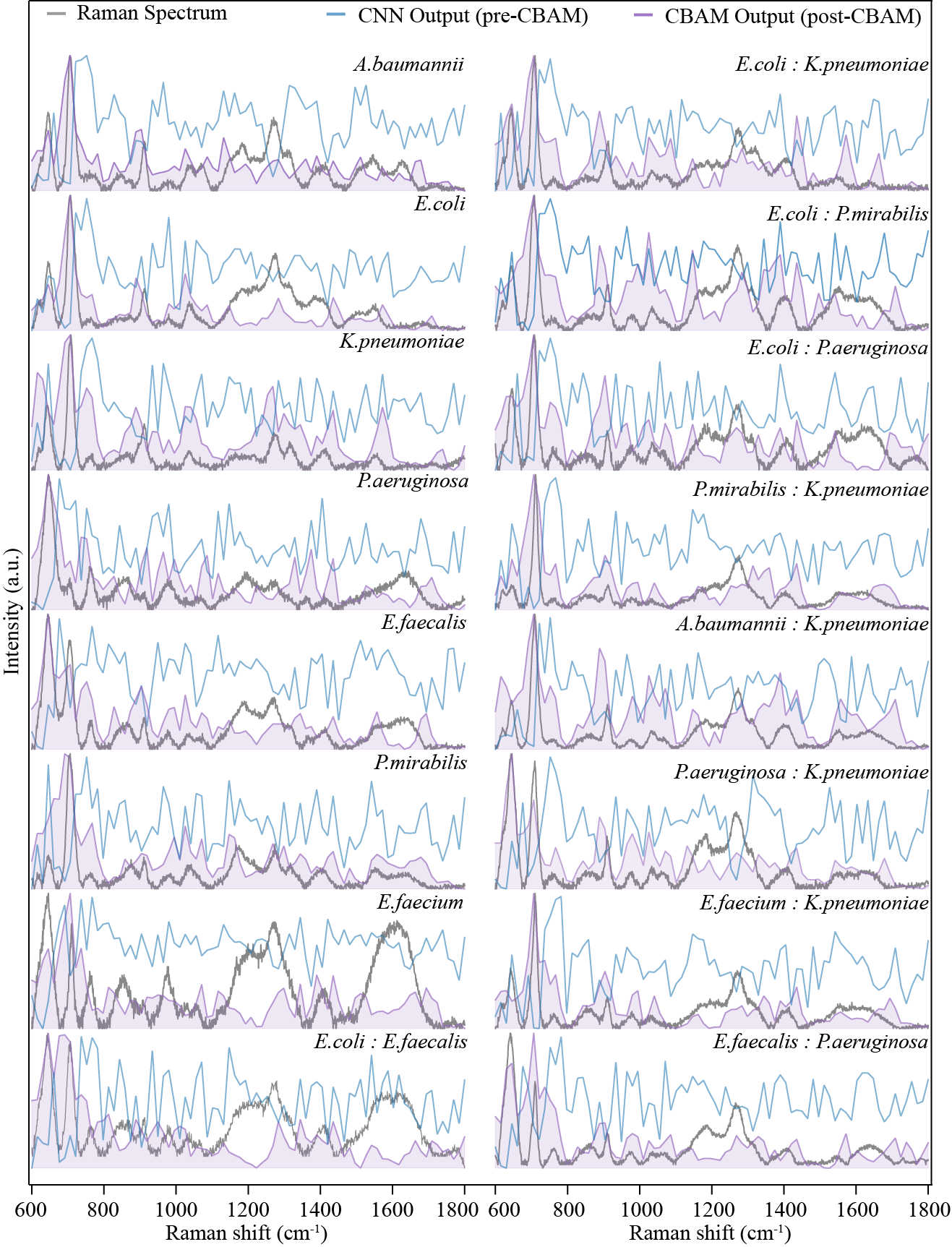


**Figure S9. Visualization of feature responses before and after CBAM enhancement for representative bacterial species and mixed infections.** The original Raman spectra (gray), CNN feature outputs before CBAM (blue), and CBAM-enhanced outputs (purple) are shown for each bacterial class.


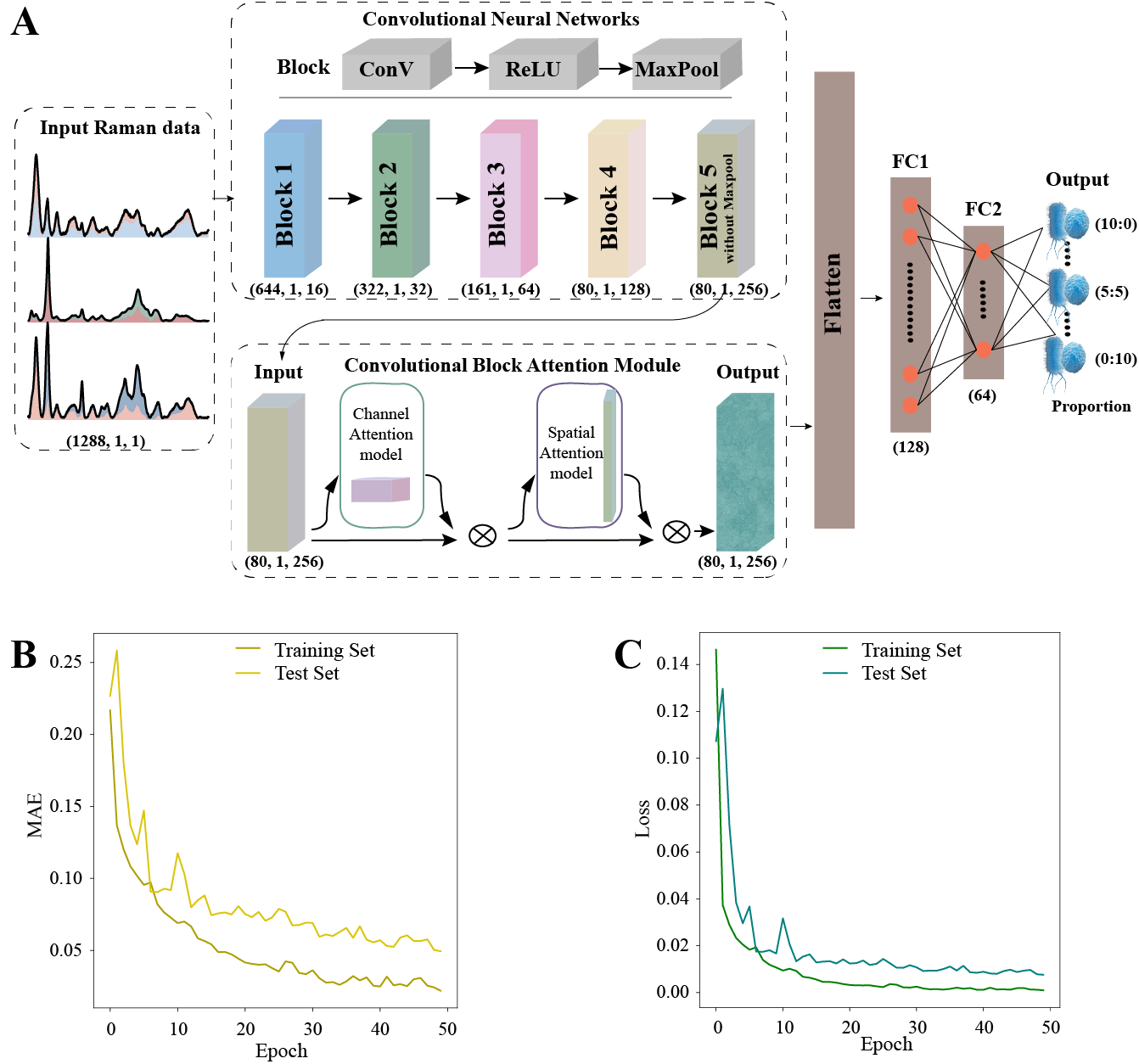


**Figure S10.** **Architecture and performance of the CNN+CBAM model for bacterial mixture ratio prediction using Raman spectra. (A)** Schematic of the convolutional neural network with a convolutional block attention module (CNN+CBAM) used for predicting the mixing ratios of two bacterial species based on Raman spectra. Mean absolute error (MAE) curves **(B)** and loss curves **(C)** for the training and test sets over 50 epochs.

**
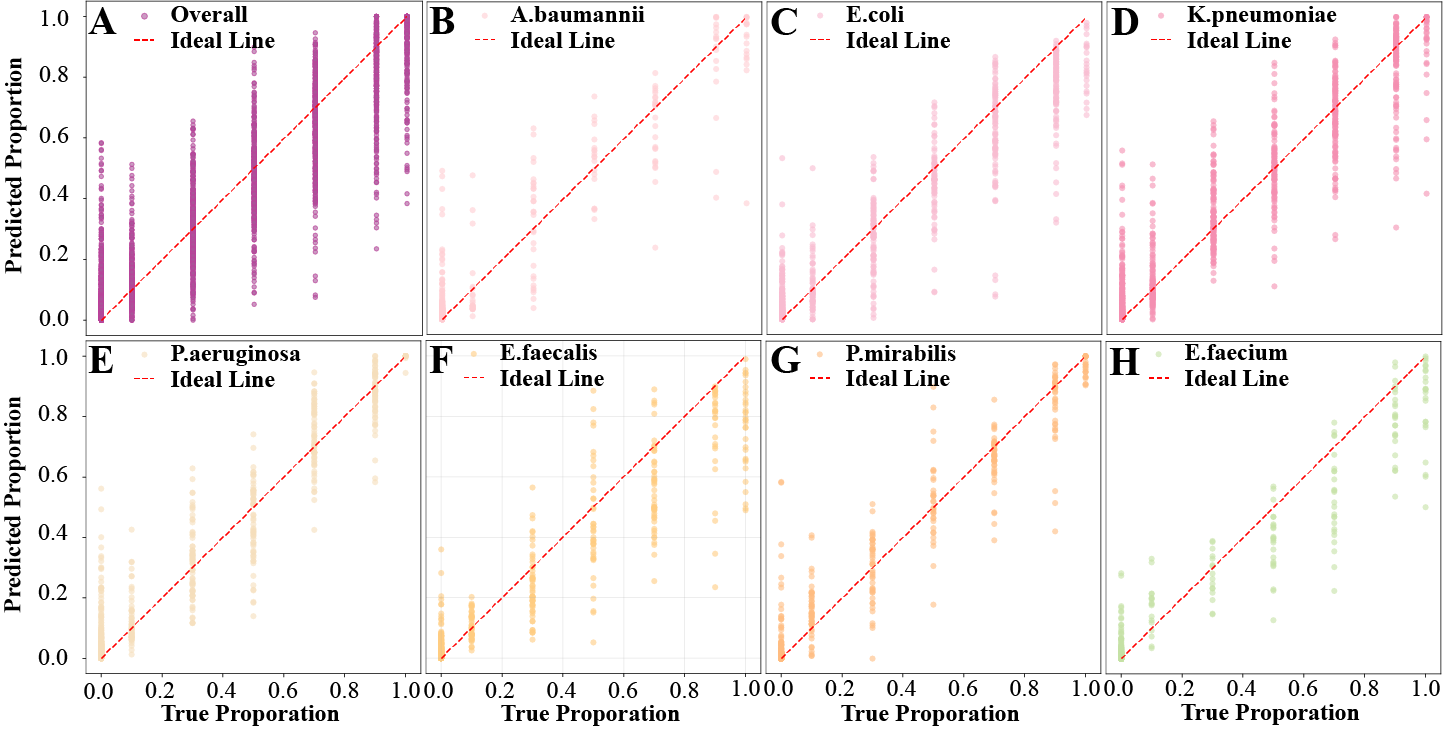
**Figure S11.** **Scatter plots comparing predicted and true proportions of individual bacterial species in binary mixtures.** (A)** Overall performance of the model in predicting bacterial proportions across all species, with each point representing a sample and the red dashed line indicating the ideal 1:1 prediction. **(B)** *A. baumannii*, **(C)** E. coli, **(D)** K. pneumoniae, **(E)** *P. aeruginosa,* **(F)** E. faecalis, **(G)** P. mirabilis, and **(H)** E. faecium. Each plot demonstrates how closely the model’s predicted values match the true mixing ratios, highlighting strong predictive performance and generalizability across diverse bacterial species.

| **Table S2**：Performance indicators of different bacterial species in proportion prediction in the CNN+CBAM model | | | |
| --- | --- | --- | --- |
| Bacteria | MSE | MAE | R^2^ |
| *A. baumannii* | 0.0041 | 0.0259 | 0.8970 |
| 1. *coli* | 0.0103 | 0.0556 | 0.9025 |
| 1. *pneumoniae* | 0.0121 | 0.0656 | 0.8922 |
| 1. *aeruginosa* | 0.0062 | 0.0467 | 0.9271 |
| 1. *faecalis* | 0.0078 | 0.0373 | 0.8879 |
| 1. *mirabilis* | 0.0037 | 0.0221 | 0.9453 |
| 1. *faecium* | 0.0036 | 0.0211 | 0.9263 |
| MSE: Mean squared error; MAE: Mean absolute error | | | |

**
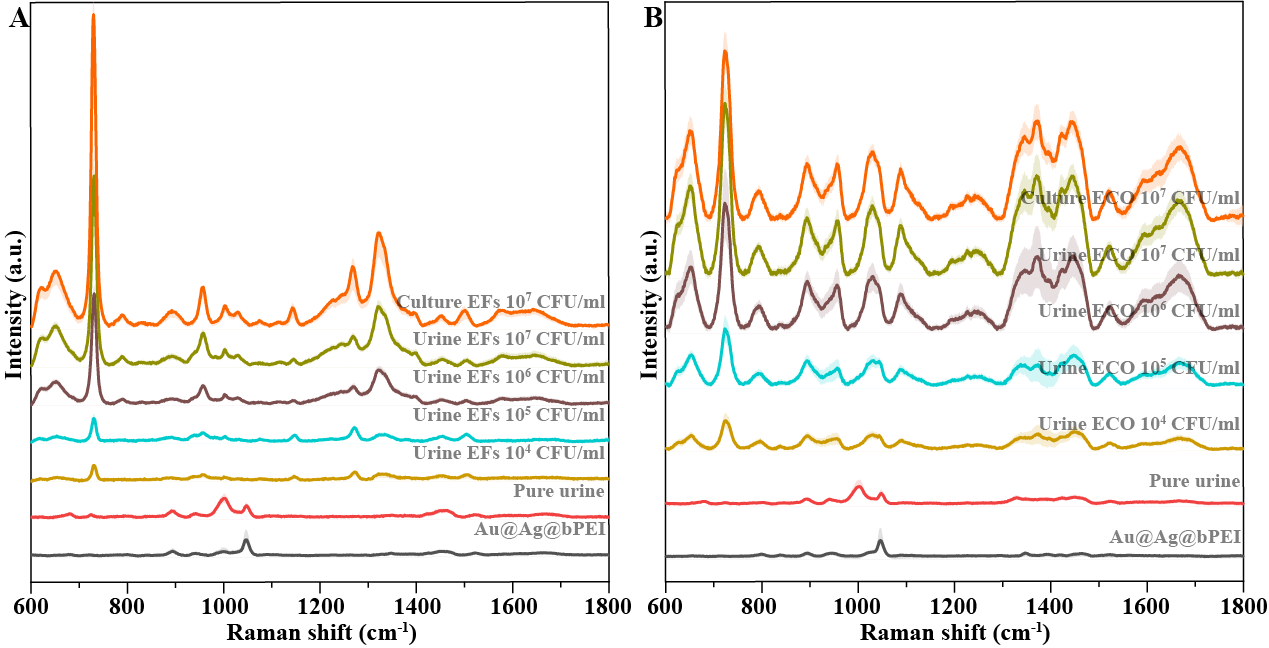
**

****Figure S12. SERS spectra of urine samples spiked with *E. faecalis* (A)** and ***E. coli* (B)** at varying concentrations, using Au@Ag@bPEI core-shell substrates.**

**To evaluate the potential influence of urine components on SERS-based bacterial detection, spectra from sterile urine, urine spiked with bacteria at varying concentrations, and pure bacterial cultures were collected. Once bacterial concentrations exceeded 10^4^ CFU/mL, the effect of the urine matrix on SERS signals became negligible, with spectral features predominantly attributable to the bacteria (**Figure**** S12**). Notably, the spectra acquired from urine-based samples were highly consistent with those obtained from pure bacterial suspensions used in the training set, indicating no significant differences.**

**
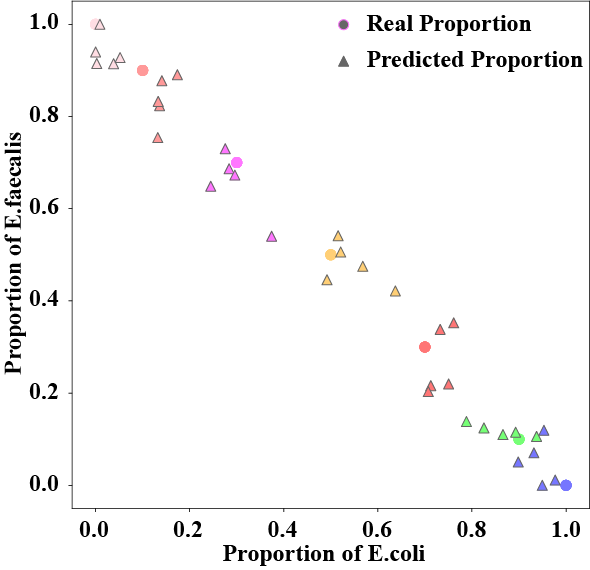
**

**Figure S13. Scatter plot comparing the real versus predicted proportions of *E. coli* and *E. faecalis* in mixed bacterial samples.**


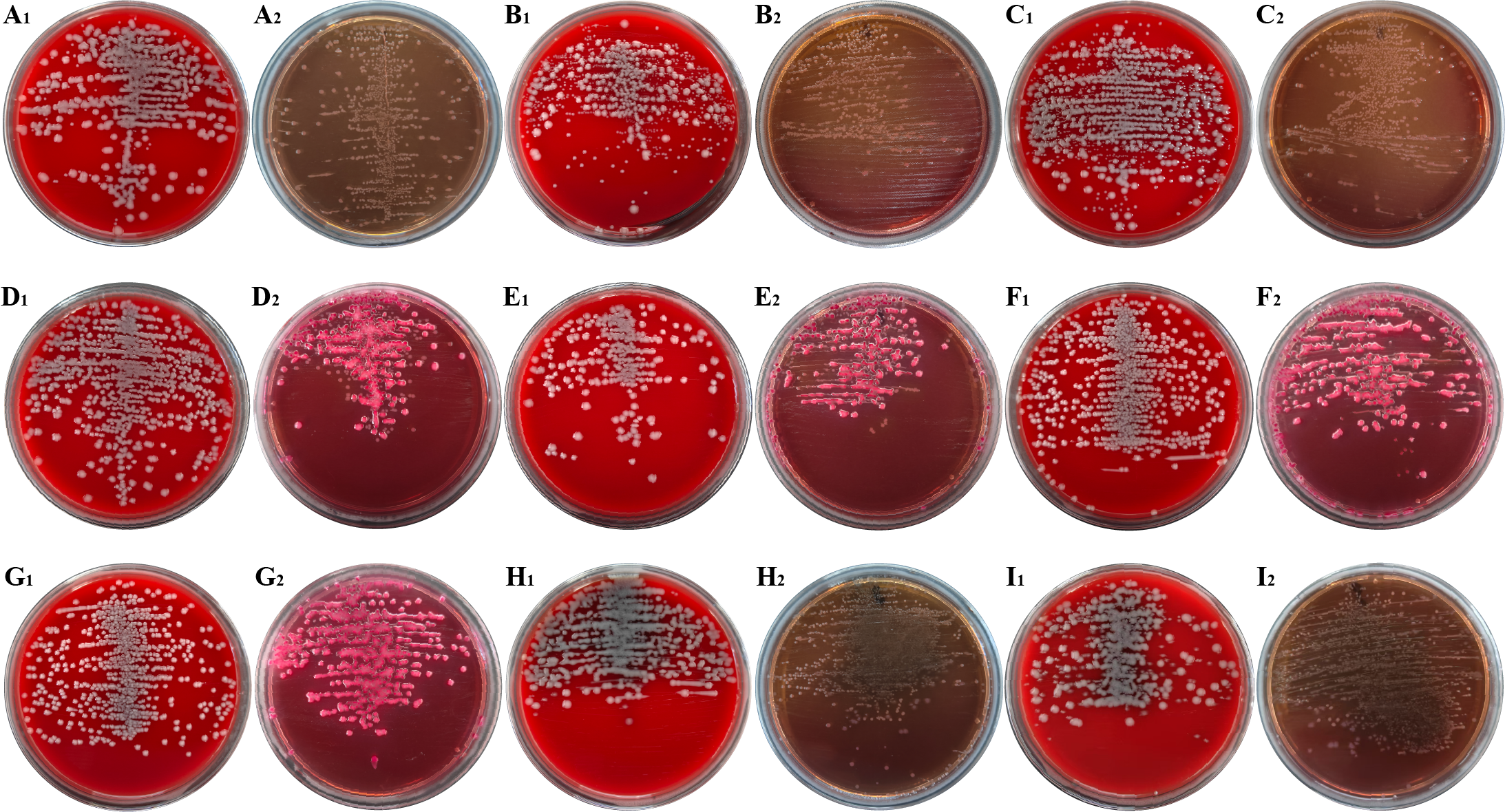


**Figure S14. Bacterial culture results from urine samples of nine patients with mixed infections.** For each patient sample, images show bacterial growth on blood agar (X_1_) and MacConkey agar plates (X_2_). The ratios shown are: (**A_1_**), (**A_2_**) *E. coli* : *E. faecalis* with 9:1; (**B_1_**) , (**B_2_**) and (**C_1_**), (**C_2_**) *E. coli* : *E. faecalis* with 5:5; (**D_1_**), (**D_2_**) and (**E_1_**), (**E_2_**) *E. coli* : *K. pneumoniae* with 5:5; (**F_1_**), (**F_2_**) *A. baumannii* : *K. pneumoniae* with 1:9; (**G_1_**), (**G_2_**) *A. baumannii* : *K. pneumoniae* with 3:7; (**H_1_**), (**H_2_**) and (**I_1_**), (**I_2_**) *E. coli* : *P. aeruginosa* with 9:1 and 5:5.

| **Table S3:** Bacterial quantification in nine patients with mixed infections | | | |
| --- | --- | --- | --- |
| Patient | Bacterial | Quantification (CFU/ml) | proportion |
| 1 | *E. coli* | 9×10^5^ | 9:1 |
|  | *E. faecalis* | 1×10^5^ |  |
| 2 | *E. coli* | 5×10^5^ | 5:5 |
|  | *E. faecalis* | 5×10^5^ |  |
| 3 | *E. coli* | 1×10^6^ | 5:5 |
|  | *E. faecalis* | 1×10^6^ |  |
| 4 | *E. coli* | 5×10^5^ | 5:5 |
|  | *K. pneumoniae* | 5×10^5^ |  |
| 5 | *E. coli* | 1×10^5^ | 5:5 |
|  | *K. pneumoniae* | 1×10^5^ |  |
| 6 | *A. baumannii* | 1×10^5^ | 1:9 |
|  | *K. pneumoniae* | 9×10^5^ |  |
| 7 | *A. baumannii* | 3×10^5^ | 3:7 |
|  | *K. pneumoniae* | 7×10^5^ |  |
| 8 | *E. coli* | 9×10^5^ | 9:1 |
|  | *P. aeruginosa* | 1×10^5^ |  |
| 9 | *E. coli* | 3×10^5^ | 5:5 |
|  | *P. aeruginosa* | 3×10^5^ |  |


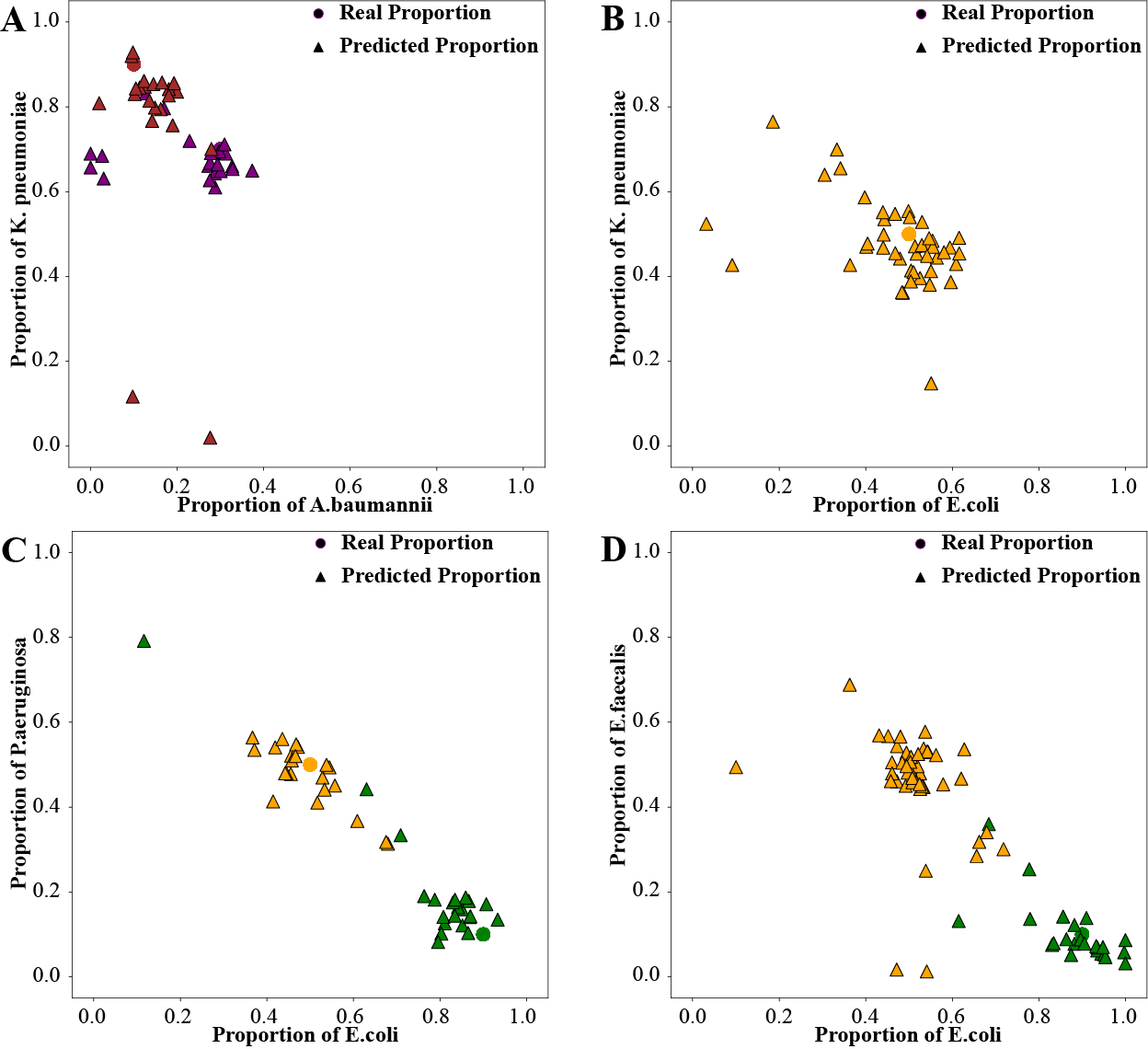


**Figure S15. Comparison between real and predicted proportions of bacterial species of mixtures in urine**. Scatter plots showing the real and predicted proportions of each bacterial species pair in different combinations. **(A)** *A. baumannii* : *K. pneumoniae*, **(B)** *E. coli* : *K. pneumoniae*, **(C)** *E. coli* : *P. aeruginosa*, and **(D)** *E. coli* : *E. faecalis*.

| **Table S4:** Comparative overview of SERS substrates, modeling approaches, and their performance for mixed bacterial species analysis | | | | | | | |
| --- | --- | --- | --- | --- | --- | --- | --- |
| No | SERS substrate | Models | mixed bacterial | | | Application | Reference |
|  |  |  | Composition | Identify | Ratio (R^2^) |  |  |
| 1 | Au@Ag NRs | PLSDA | *E. coli with* *S. aureus*；  *S. aureus* with *B. subtilis*;  *E. coli* with *P. aeruginosa* | NA | NA | NA | [23] |
| 2 | Ag NRs | PCA | *E.coli with* *S. aureus*；  *K. pneumonia* with *P. aeruginosa* | - | NA | NA | [23] |
| 3 | Au@AgNPs | - | *E. coli with B.subtilis* | NA | NA | Yam slices  NA | [23] |
| 4 | Au@Ag@SiO2 | SNV-  PLSDA | *E. coli with S. aureus*;  *E. coli with S. typ*;  S. aureus *with S. typ*;  *S. aureus*, *E. coli with S. typ* | 92% | 0.904-0.966 | NA | [23] |
| 5 | Au@Ag@SiO2 | ANNs | *E. coli with* *S. aureus*;  *E. coli with S. typ*;  *S. aureus with S. typ;*  *S. aureus* with *E. coli with S. typ* | - | 0.954-0.981 | NA | [23] |
| 6 | Au NPs | - | *E. coli* with *P. aeruginosa* | imaging analysis | - | NA | [23] |
| 7 | CaF2 | PCA-  LDA | *E. coli with E. faecalis;*  *E. coli with S. warneri;*  *E. coli with S. aureus* | 87% | - | Artiﬁcial mixtures  75% | [23] |
| 8 | Au NRs | RF | *S. epidermidis* with *E. coli* | 92% | - | Blood  87% | [23] |
| 9 | NA | SVM | *E. coli* with *S. aureus*;  *E. coli*, *S. aureus* with *P. vulgaris* | 89% | - | NA | [23] |
| 10 | Au NPs | PCA | *L.mon* with *S.aureus*  *L.mon* with *E.coli*  *L.mon* with *S.enteritidis*  *E.coli* with *S.enteritidis*  *E.coli* with *S.aureus*  *S.aureus* with *S.enteritidis* | NA | - | NA | [23] |
| 11 | Au@Ag@bPEI | CNN-  CBAM | *E. coli* with *E. faecalis;*  *E. coli* with *K. pneumoniae;*  *A. baumannii* with *K. pneumoniae;*  *E. coli* with *P. aeruginosa;*  *E. coli* with *P. mirabilis;*  *E. faecium* with *K. pneumoniae;*  *E. faecalis* with *P. aeruginosa;*  *P. mirabilis* with *K. pneumoniae;*  *P. aeruginosa* with *K. pneumoniae* | 95.8% | 0.911 | Urine  Accuracy = 86.9%；  R² = 0.8626 | This study |
| NRs: Nanorods; PLS-DA: Partial least squares discrimination analysis; PCA: Principal component analysis; SNV: Standard normal variate; ANN: Artiffcial neural networks; LDA: Linear discriminant analysis; RF: Random forest; SVM: Support vector machine; CBAM: Convolutional block attention module; *E. coli*: *Escherichia coli*; *S. aureus*: *Staphylococcus aureus*; *B. subtilis*: *Bacillus subtilis*; *P. aeruginosa*: *Pseudomonas aeruginosa*; *K. pneumonia*: *Klebsiella pneumoniae*; *S. typ*: *Salmonella typhimurium*; *E. faecalis: Enterococcus faecalis; S. warneri: Staphylococcus warneri; S. epidermidis: Staphylococcus epidermidis; P. vulgaris*: *Proteus vulgaris*; *L. mon: Listeria monocytogenes; S. enteritidis: Salmonella enteritidis; A. baumannii: Acinetobacter baumannii; P. mirabilis: Proteus mirabilis*; *E. faecium*: *Enterococcus faecium;* NA: Not available. | | | | | | | |

**Reference:**

1. Y. Xu, W. Aljuhani, Y. Zhang, Z. Ye, C. Li, S. Bell, Chem Soc Rev 2025, 54, 62.

2. H. Wang, Y. Zhou, X. Jiang, B. Sun, Y. Zhu, H. Wang, Y. Su, Y. He, Angew Chem Int Ed Engl 2015, 54, 5132.

3. X. Zhao, Y. Wang, Y. Yao, L. Chen, B. Lin, W. Zheng, Y. Zeng, L. Li, Y. She, L. Guo, Anal Chem 2023, 95, 6836.

4. K. Maquelin, C. Kirschner, L. P. Choo-Smith, N. van den Braak, H. P. Endtz, D. Naumann, G. J. Puppels, J Microbiol Methods 2002, 51, 255.

5. S. Das, K. Saxena, J. C. Tinguely, A. Pal, N. L. Wickramasinghe, A. Khezri, V. Dubey, A. Ahmad, V. Perumal, R. Ahmad, D. N. Wadduwage, B. S. Ahluwalia, D. S. Mehta, ACS Appl Mater Interfaces 2023, 15, 24047.

6. Z. Liu, C. Davis, W. Cai, L. He, X. Chen, H. Dai, Proc Natl Acad Sci U S A 2008, 105, 1410.

7. E. Papadopoulou, S. E. Bell, Chemistry 2012, 18, 5394.

8. G. Shetty, C. Kendall, N. Shepherd, N. Stone, H. Barr, Br J Cancer 2006, 94, 1460.

9. A. J. Ruiz-Chica, M. A. Medina, F. Sanchez-Jimenez, F. J. Ramirez, J Raman Spectros 2004, 35, 93.

10. H. Nawaz, F. Bonnier, A. D. Meade, F. M. Lyng, H. J. Byrne, Analyst 2011, 136, 2450.

11. W. T. Cheng, M. T. Liu, H. N. Liu, S. Y. Lin, Microsc Res Tech 2005, 68, 75.

12. A. Walter, A. Marz, W. Schumacher, P. Rosch, J. Popp, Lab Chip 2011, 11, 1013.

13. T. Bhattacharjee, P. Kumar, G. Maru, A. Ingle, C. M. Krishna, Lasers Med Sci 2014, 29, 325.

14. F. Bonnier, H. J. Byrne, Analyst 2012, 137, 322.

15. W. A. Asfaw, K. D. Tafa, N. Satheesh, Heliyon 2023, 9, e13724.

16. Y. Chen, J. Dai, X. Zhou, Y. Liu, W. Zhang, G. Peng, PLoS One 2014, 9, e93906.

17. L. M. Almond, J. Hutchings, G. Lloyd, H. Barr, N. Shepherd, J. Day, O. Stevens, S. Sanders, M. Wadley, N. Stone, C. Kendall, Gastrointest Endosc 2014, 79, 37.

18. A. J. Ruiz Chica, M. A. Medina, F. Sánchez Jiménez, F. J. Ramírez, Journal of Raman Spectroscopy 2004, 35, 93.

19. L. R. Jyothi, V. B. Kartha, K. C. Murali, S. J. R, G. Ullas, D. P. Uma, Radiat Res 2002, 157, 175.

20. S. Farquharson, C. Shende, F. E. Inscore, P. Maksymiuk, A. Gift, Journal of Raman Spectroscopy 2005, 36, 208.

21. Y. Wang, K. Lee, J. Irudayaraj, The Journal of Physical Chemistry C 2010, 114, 16122.

22. H. Liang, X. Kong, Y. Ren, H. Wang, E. Liu, F. Sun, G. Zhu, Q. Zhang, Y. Zhou, Spectrochim Acta A Mol Biomol Spectrosc 2023, 302, 123008.

23. D. P. Lau, Z. Huang, H. Lui, D. W. Anderson, K. Berean, M. D. Morrison, L. Shen, H. Zeng, Lasers Surg Med 2005, 37, 192.

24. K. Shirota, K. Yagi, T. Inaba, P. C. Li, M. Murata, Y. Sugita, T. Kobayashi, Biophys J 2016, 111, 999.

25. N. Stone, C. Kendall, J. Smith, P. Crow, H. Barr, Faraday Discuss 2004, 126, 141, 169.

26. S. Singh, T. Verma, B. Khamari, E. P. Bulagonda, D. Nandi, S. Umapathy, Anal Chem 2023, 95, 11342.
